# A structurally derived model of subunit‐dependent NMDA receptor function

**DOI:** 10.1113/JP276093

**Published:** 2018-08-01

**Authors:** Alasdair J. Gibb, Kevin K. Ogden, Miranda J. McDaniel, Katie M. Vance, Steven A. Kell, Chris Butch, Pieter Burger, Dennis C. Liotta, Stephen F. Traynelis

**Affiliations:** ^1^ Department of Neuroscience Physiology and Pharmacology University College London Gower Street London WC1E 6BT UK; ^2^ Department of Pharmacology Emory University School of Medicine Rollins Research Center 1510 Clifton Road Atlanta GA 30322 USA; ^3^ Department of Chemistry Emory University School 1515 Dickey Drive Atlanta GA 30322 USA

**Keywords:** NMDA receptor, Synaptic mechanisms, modelling, Activation kinetics

## Abstract

**Key points:**

- The kinetics of NMDA receptor (NMDAR) signalling are a critical aspect of the physiology of excitatory synaptic transmission in the brain.
- Here we develop a mechanistic description of NMDAR function based on the receptor tetrameric structure and the principle that each agonist‐bound subunit must undergo some rate‐limiting conformational change after agonist binding, prior to channel opening.
- By fitting this mechanism to single channel data using a new MATLAB‐based software implementation of maximum likelihood fitting with correction for limited time resolution, rate constants were derived for this mechanism that reflect distinct structural changes and predict the properties of macroscopic and synaptic NMDAR currents.
- The principles applied here to develop a mechanistic description of the heterotetrameric NMDAR, and the software used in this analysis, can be equally applied to other heterotetrameric glutamate receptors, providing a unifying mechanistic framework to understanding the physiology of glutamate receptor signalling in the brain.

**Abstract:**

NMDA receptors (NMDARs) are tetrameric complexes comprising two glycine‐binding GluN1 and two glutamate‐binding GluN2 subunits. Four GluN2 subunits encoded by different genes can produce up to 10 different di‐ and triheteromeric receptors. In addition, some neurological patients contain a *de novo* mutation or inherited rare variant in only one subunit. There is currently no mechanistic framework to describe tetrameric receptor function that can be extended to receptors with two different GluN1 or GluN2 subunits. Here we use the structural features of glutamate receptors to develop a mechanism describing both single channel and macroscopic NMDAR currents. We propose that each agonist‐bound subunit undergoes some rate‐limiting conformational change after agonist binding, prior to channel opening. We hypothesize that this conformational change occurs within a triad of interactions between a short helix preceding the M1 transmembrane helix, the highly conserved M3 motif encoded by the residues SYTANLAAF, and the linker preceding the M4 transmembrane helix of the adjacent subunit. Molecular dynamics simulations suggest that pre‐M1 helix motion is uncorrelated between subunits, which we interpret to suggest independent subunit‐specific conformational changes may influence these pre‐gating steps. According to this interpretation, these conformational changes are the main determinants of the key kinetic properties of NMDA receptor activation following agonist binding, and so these steps sculpt their physiological role. We show that this structurally derived tetrameric model describes both single channel and macroscopic data, giving a new approach to interpreting functional properties of synaptic NMDARs that provides a logical framework to understanding receptors with non‐identical subunits.

## Key points

- The kinetics of NMDA receptor (NMDAR) signalling are a critical aspect of the physiology of excitatory synaptic transmission in the brain.
- Here we develop a mechanistic description of NMDAR function based on the receptor tetrameric structure and the principle that each agonist‐bound subunit must undergo some rate‐limiting conformational change after agonist binding, prior to channel opening.
- By fitting this mechanism to single channel data using a new MATLAB‐based software implementation of maximum likelihood fitting with correction for limited time resolution, rate constants were derived for this mechanism that reflect distinct structural changes and predict the properties of macroscopic and synaptic NMDAR currents.
- The principles applied here to develop a mechanistic description of the heterotetrameric NMDAR, and the software used in this analysis, can be equally applied to other heterotetrameric glutamate receptors, providing a unifying mechanistic framework to understanding the physiology of glutamate receptor signalling in the brain.

## Introduction

*N*‐Methyl‐d‐aspartate (NMDA) receptors are a subtype of ionotropic glutamate receptor with important roles in excitatory synaptic transmission, synaptic plasticity, learning and memory (Bliss & Collingridge, 1993; Cull‐Candy & Leszkiewicz, 2004; Traynelis *et al*. 2010; Paoletti *et al*. 2013). Their role as coincidence detectors and in spike timing‐dependent plasticity (Markram *et al*. 1997, 2012; Nevian & Sakmann, 2004; Froemke *et al*. 2010; Hao & Oertner, 2012) makes the time course of the synaptic NMDA receptor response an important determinant of circuit function in the central nervous system. Thus, insight into receptor function will enhance our understanding of excitatory synaptic transmission, as well as a wide range of processes in the brain. NMDA receptors are also implicated in multiple neurological diseases (Choi, 1992; Palmer, 2001; Hallett & Standeart, 2004; Ross *et al*. 2006; Preskorn *et al*. 2008, 2015; Kantrowitz & Javitt, 2010; Coyle, 2012; Paoletti *et al*. 2013; Parsons & Raymond, 2014; Yuan *et al*. 2015; Hu *et al*. 2016), and thus are a target for new therapeutically relevant classes of allosteric modulators that are currently being developed (Bettini *et al*. 2010; Costa *et al*. 2010; Mosley *et al*. 2010; Mullasseril *et al*. 2010; Acker *et al*. 2011; Ogden & Traynelis, 2013; Santangelo Freel *et al*. 2013; Khatri *et al*. 2014; Hackos *et al*. 2016; Strong *et al*. 2017; Swanger *et al*. 2018).

### Receptor structure and subunit stoichiometry

Recent structural data (Karakas & Furukawa, 2014; Lee *et al*. 2014; Tajima *et al*. 2016; Zhu *et al*. 2016; Lü *et al*. 2017; Mesbahi‐Vasey *et al*. 2017) have yielded insight into the functional contributions of multiple domains within each subunit. The structural evidence emerging from these studies are complementary to many functional studies (Gielen et al., 2008, 2009; Yuan *et al*. 2009; Talukder *et al*. 2010; Talukder & Wollmuth, 2011; Zhu *et al*. 2013; Kazi *et al*. 2013, 2014; Borschel *et al*. 2015; Glasgow *et al*. 2015; Mayer, 2017) and can be used to inform new models of NMDA receptor function. For example, the NMDA receptor has twofold symmetry throughout its extracellular domain, dominated by heterodimerization of the GluN1/GluN2 amino terminal domains as well as the GluN1/GluN2 ligand binding domains. The interfaces within and between these heterodimers provide strong inter‐subunit and inter‐domain dependence to agonist binding (Mayer *et al*. 1989; Benveniste *et al*. 1990; Kew *et al*. 1996; Zheng *et al*. 2001) and define important new modulator binding sites (Karakas *et al*. 2011; Hansen *et al*. 2012; Hackos *et al*. 2016; Stroebel *et al*. 2016). The receptor complex undergoes a transition to fourfold symmetry approaching the ion channel pore, where linker regions connect the ligand binding domains to the highly conserved transmembrane domains (Karakas & Furukawa, 2014; Lee *et al*. 2014). This region includes the signature M3 gating motif (SYTANLAAF) as well as a critically positioned two‐turn cuff helix that lies parallel to the plasma membrane and is in van der Waals contact with the M3 transmembrane helix that occludes the pore in the closed state (Sobolevsky *et al*. 2009; Karakas *et al*. 2015; Tajima *et al*. 2016). Receptors composed of two identical GluN1 and two identical GluN2 subunits likely show equivalence in the rates of conformational change within subunit dimer pairs, while receptors with non‐identical GluN1 or GluN2 subunits (often referred to as triheteromeric receptors) will lack this symmetry (Lü *et al*. 2017).

### Receptor activation

Within each subunit, conformational changes in the extracellular domains involve a closure of a bi‐lobed structure around glycine for GluN1 (Furukawa & Gouaux, 2003; Inanobe *et al*. 2005) and glutamate for GluN2 (Furukawa *et al*. 2005; Vance *et al*. 2011; Hansen *et al*. 2013). Several studies support the idea that NMDA receptors must bind two glutamate and two glycine molecules before they can open (Benveniste & Mayer, 1991; Clements & Westbrook, 1991). This is followed by at least two kinetically distinct conformational changes that precede extremely rapid opening of the ion channel (Banke & Traynelis, 2003; Schorge *et al*. 2005). The existence of these kinetically distinct conformations has been inferred from multiple components of the single channel current closed time distributions (Howe *et al*. 1991; Gibb & Colquhoun, 1992; Wyllie *et al*. 1998) as well as maximum likelihood fitting of recordings from a single active channel with kinetic models (Banke & Traynelis, 2003; Popescu *et al*. 2004; Auerbach & Zhou, 2005; Erreger *et al*. 2005; Schorge *et al*. 2005). The rates of these conformational changes logically must depend on the identity of the GluN1 and GluN2 subunits, and may reflect conformational changes within each subunit (Banke & Traynelis, 2003; Erreger *et al*. 2005) or concerted channel motions linking extracellular and transmembrane domains, such as a vertical movement of GluN1 pre‐M3 linker and the lateral separation of GluN2B pre‐M3 linker by 7 Å and 11 Å, respectively (Dai & Zhou, 2013; Tajima *et al*. 2016). These pre‐gating steps do not reflect the process of agonist‐induced closure of the bi‐lobed glutamate binding domain, as glutamate binding must occur on a sub‐millisecond time scale since responses to 1–4 ms application of a maximally effective concentration of glutamate produce a maximal response (Banke & Traynelis, 2003), indicating glutamate binding (including closure of the clamshell domain) is complete within a millisecond. Once the multimeric receptor has traversed these subunit‐dependent pre‐gating steps, channel opening likely occurs rapidly (with a rate too fast to measure) and involves dilatation of the gating ring by coordinated rearrangement of the M3 helical bundle crossing, which occludes the ion conducting pore in the resting state (Karakas *et al*. 2015; Tajima *et al*. 2016). A bend in the M3 helix within the SYTANLAAF motif just above the two‐turn pre‐M1 helix also may occur in response to tension produced by closure of the bi‐lobed agonist binding domain (Twomey & Sobolevsky, 2017; Twomey *et al*. 2017). Structural features of the receptor that control these functionally inferred pre‐gating conformational changes are currently unknown, but recent insights from disease‐causing mutations (Yuan *et al*. 2014; Chen *et al*. 2017; Ogden *et al*. 2017) are beginning to highlight specific regions of the protein as candidates for gating elements.

### Triheteromeric NMDA receptors

There is a wealth of detailed functional studies of diheteromeric NMDA receptors that contain two identical GluN1 and two identical GluN2 subunits in the literature (Traynelis *et al*. 2010; Paoletti *et al*. 2013). However, several lines of evidence suggest that NMDA receptors often contain two different GluN2 subunits (Sheng *et al*. 1994; Chazot & Stephenson, 1997; Dunah *et al*. 1998; Rauner & Köhr, 2011; Delaney *et al*. 2013; Tovar *et al*. 2013). For example, the majority of synaptic NMDA receptors in the hippocampus contain one GluN2A and one GluN2B subunit (Rauner & Köhr, 2011; Tovar *et al*. 2013), and cerebellar Golgi cells, subthalamic nucleus and substantia nigra neurons express NMDA receptors with properties consistent with one GluN2B and one GluN2D subunit (Brickley *et al*. 2003; Jones & Gibb, 2005; Brothwell *et al*. 2008; Huang & Gibb, 2014; Swanger *et al*. 2015).

Recent advances in techniques to express receptors that contain two different GluN2 subunits in heterologous expression systems (Hatton & Paoletti, 2005; Hansen *et al*. 2014; Stroebel *et al*. 2014) reveal surprising properties for these triheteromeric receptors, such as substantial residual currents in saturating concentration of subunit‐selective allosteric antagonists, which cannot be explained by existing models of NMDA receptor function. Moreover, triheteromeric receptors have been shown to display kinetic activation and deactivation properties that are dominated by a single GluN2 subunit, with properties distinct from either diheteromeric receptor (Hansen *et al*. 2014; Sun *et al*. 2017). Finally, a large number of *de novo GRIN1*, *GRIN2A* and *GRIN2B* mutations exist in patients that are heterozygous for the affected allele (Yuan *et al*. 2015; Hu *et al*. 2016), increasing the likelihood that receptors will contain one wild type and one mutant NMDAR subunit. Thus, there is a need to develop models that explicitly identify individual receptor subunits in order to enable the evaluation of the mechanisms by which multimeric receptors with two different GluN1 or GluN2 subunits function, so that the synaptic and pharmacological properties of these receptors can be understood and exploited for therapeutic gain.

### Mechanisms describing NMDA receptor activation

NMDA receptor activation mechanisms have been developed to describe both the slow rise and decay time course of synaptic currents (Lester & Jahr, 1992) as well as the greater complexities evident on analysing single channel data (Banke & Traynelis, 2003; Popescu *et al*. 2004; Auerbach & Zhou, 2005; Schorge *et al*. 2005). Here we have developed an approach to analyse the functional properties of receptors that explicitly represents each GluN1 and GluN2 subunit within a single diheteromeric tetrameric complex, which allows a logical progression to modelling receptors that contain two different GluN1 or GluN2 subunits, two different GluN1 splice variants or one copy of a disease‐associated *de novo* mutation. The approach relies on insight into structural elements provided by atomic level data as well as function‐altering mutations identified in human patients. The models derived from this insight represent transitions between conformational states that are compatible with existing structural data.

We use this approach to study single channel function of diheteromeric GluN1/GluN2A receptors, because they have been extensively studied at both macroscopic and single channel level (Wyllie *et al*. 1998, Popescu & Auerbach, 2003; Popescu *et al*. 2004; Auerbach & Zhou, 2005; Erreger & Traynelis, 2005, 2008; Erreger *et al*. 2005, 2007; Schorge *et al*. 2005; Wyllie *et al*. 2006; Kazi et al., 2013, 2014) and because GluN2A is a locus for disease‐associated human mutations (Endele *et al*. 2010; Lemke *et al*. 2013; Lesca *et al*. 2013). Our analyses confirm that our model can account for wild type GluN1/GluN2A receptor synaptic function.

## Methods

### Maintenance of HEK 293 cells

HEK 293 cells (ATCC CRL 1573, Rockville, MD, USA; hereafter HEK cells) were maintained in humidified 5% CO_2_ (at 37 °C) in Dulbecco's modified Eagle's medium with GlutaMax (Invitrogen, Carlsbad, CA, USA) supplemented with 10% dialysed fetal bovine serum, 10 μg ml^−1^ streptomycin, and 10 units ml^−1^ penicillin, as previously described (Yuan *et al*. 2009). Cells were trypsinized and plated onto glass coverslips (Warner Instruments, Hamden, CT, USA) coated with poly‐d‐lysine at 100 μg ml^−1^ for single channel and 5 μg ml^−1^ for whole‐cell recordings. Coverslips were transferred to 24‐well plates with 0.5 ml of supplemented media in each well.

### Single channel recording and analysis

Cells were transfected using FuGENE 6 (Roche Diagnostics, Basel, Switzerland) with cDNA encoding green fluorescent protein (GFP), wild type GluN1‐1a (GenBank accession numbers U11418 and U08261; hereafter GluN1), and GluN2A (D13211) and GluN2B (U11419) at a ratio of 5:1:1 (GFP:GluN1:GluN2); total cDNA was 0.2 mg ml^−1^. All rat NMDA receptor subunit cDNAs were provided by Drs Heinemann (Salk Institute) and Nakanishi (Kyoto University). After transfection, the HEK cells were incubated for 12–24 h (timed to optimize observation of patches with a single active channel) in media supplemented with d,l‐2‐amino‐5‐phosphonovalerate (dl‐AP‐5) and 7‐chlorokynurenic acid (both at 200 μM) prior to patch clamp recordings.

Single channel recordings were made from excised outside‐out patches with an Axopatch 200B amplifier (Molecular Devices, Union City, CA, USA) at room temperature (23 °C) using thick‐wall glass pipettes (G150F‐4, Warner Instruments Inc., Hamden, CT, USA) coated with heat‐cured Sylgard (Dow Corning, Midland, MI, USA). Outside‐out patch recordings were chosen for these studies to reduce the occurrence of modal behaviour (Popescu & Auerbach, 2003) within the recording. Recording pipettes were fire‐polished to a final resistance of 8.5–11 ΜΩ. The holding potential was −80 mV. The internal (pipette) solution contained (in mM) 110 d‐gluconate, 110 CsOH, 30 CsCl, 5 HEPES, 4 NaCl, 0.5 CaCl_2_, 2 MgCl_2_, 5 BAPTA, 2 Na‐ATP, and 0.3 Na‐GTP (pH 7.35); the osmolality was adjusted to 300–310 mosmol kg^−1^ using CsCl or water. The external solution contained (in mM) 150 NaCl, 3 KCl, 10 HEPES, 30 d‐mannitol, 0.5 CaCl_2_, and 0.01 EDTA at pH 8.0. Recombinant GluN1/GluN2A channels in patches were activated by addition of 50 μM glycine and 1 mM glutamate to the external solution. The steady state response of channels was recorded during continuous perfusion of the agonist‐supplemented solution. Voltage‐clamp recordings were filtered at 8 kHz (−3 dB Bessel) and digitized at 40 kHz (pCLAMP 9). Current recordings were analysed off‐line following digital filtering at 4 kHz, and channel open and closed times determined from the idealization of the record by the time course fitting method using a step response function filtered at 4 kHz (SCAN software provided by Professor David Colquhoun, University College London, http://www.ucl.ac.uk/Pharmacology/dcpr95.html).

Before a patch recording was accepted for detailed analysis, the long‐term stability of the data record was checked for mode changes that could complicate model fitting (Blatz & Magleby, 1989; Colquhoun & Sigworth, 1995; Popescu & Auerbach, 2003) by making stability plots for amplitudes longer than 2.0 filter rise times, open times, closed times and open probability (*P*_open_) (Colquhoun & Sigworth, 1995). If the record was deemed stable, distributions of channel open times or closed times were fitted using the maximum likelihood method with probability density functions that were a sum of two exponential components for open times and five exponential components for closed times (Gibb & Colquhoun, 1992; Wyllie *et al*. 1998; Popescu *et al*. 2004; Erreger *et al*. 2005; Schorge *et al*. 2005; EKDIST, http://www.ucl.ac.uk/Pharmacology/dcpr95.html). Bursts of channel openings were defined using a critical closed time, t‐crit, which minimizes the number of long closed events that are misclassified as within rather than between bursts (Colquhoun & Sigworth, 1995). These analyses allowed selection of a set of low noise patches with stable and homogeneous properties. The number of channels in the patch was determined statistically as previously described (Colquhoun & Hawkes, 1990; Dravid *et al*. 2008). Only recordings with a low probability (*P* < 0.001) of containing more than one active channel were used for data analysis. The open and shut time properties for these four patches have been previously reported (Yuan *et al*. 2009).

### Macroscopic current recording and analysis

All GluN2 cDNAs used for whole cell current recordings encoded a fusion protein containing the GluN2A C‐terminal domain fused to coiled‐coil C1 and C2 peptide tags followed by an endoplasmic reticulum (ER) retention signal fused in frame to the C‐terminal. This allowed control of the stoichiometry of GluN2 in surface receptors, as previously described (Hansen *et al*. 2014). The vector containing GluN1 also independently expressed GFP and was provided by Dr Kasper Hansen (University of Montana). HEK cells were maintained and plated onto glass coverslips coated with poly‐d‐lysine (5 μg ml^−1^) as described above and were transiently transfected using the calcium phosphate precipitation method with plasmid cDNAs encoding wild type GluN1/GluN2A_C1_/GluN2A_C2_, GluN1/GluN2B_C1_/GluN2B_C2_, or GluN1/GluN2A_C1_/GluN2B_C2_ as previously described (Chen & Okayama, 1987). NMDAR subunit cDNAs were co‐transfected with cDNAs at a ratio of 1.5:1:1:0.5 for GluN1:GluN2_C1_:GluN2_C2_:GFP (0.2 mg ml^−1^ total cDNA). dl‐AP‐5, 200 μM, and 7‐chlorokynurenic acid, 200 μM, were included in the culture media to prevent excessive NMDAR activation and to reduce excitotoxic cell death. Eighteen to 24 hours following transfection, coverslips containing HEK cells were transferred to a recording chamber and continuously perfused at 2 ml min^−1^ with the recording solution described above except with d‐mannitol reduced to 11 mM and pH adjusted to 7.4 by addition of NaOH. Microelectrodes were fabricated using thin‐walled filamented borosilicate glass (World Precision Instruments cat. no. TW150F‐4) pulled using a Flaming–Brown puller (Sutter Instrument P‐1000, Hitchin, Hertfordshire, UK) and filled with the same internal recording solution described above. Pipettes filled with internal solution had resistances of 3–4 MΩ when placed into the recording solution. The membrane potential of HEK cells was held at −40 mV using an Axopatch 200B patch‐clamp amplifier (Molecular Devices) and NMDAR current responses to brief (5 ms) or prolonged (1 s) rapid external application of glutamate (1 mM) and glycine (30 μM) were recorded at room temperature (23 °C), filtered at 8 kHz (−3 dB, 8 pole Bessel filter, Frequency Devices) and digitized at 20 kHz using a Digidata 1440A data acquisition system (Molecular Devices) controlled by Clampex 10.3 (Molecular Devices). Junction potential currents were used to confirm the duration of the brief application of agonist. The current response deactivation time course was fitted to the sum of one or two exponential functions using non‐linear least squares fitting in ChanneLab (Synaptosoft, Decatur, GA, USA).

To determine the macroscopic current time course, outside‐out patches were excised and placed in front of the rapid agonist application system, and responses recorded to application of 0.003 or 1 mM glutamate and 100 μM glycine. Recording solutions and pipettes were identical to those used to record single channels in outside‐out patches. At the end of the experiment, the patch was destroyed by applying pressure and the junction potential recorded by diluting the glutamate solution 25% to determine the duration of agonist application. Waveforms were averaged across patches and normalized to the peak response to maximally effective concentrations of glutamate and glycine.

### Fitting of structure‐based mechanisms to the single channel data

Single channel openings were idealized by fitting a filtered step response function (4 kHz) to each instance of channel opening and closing to identify the correct interval and amplitude as described above. Conceptual models of channel function consisted of a set of rate constants representing the rates for transitions between hypothetical conformations proposed from structural information. Three different software implementations of the maximum likelihood fitting method with corrections for missed events due to recording bandwidth were used to evaluate the fit of different mechanisms to the idealized data: MIL (QuB software, http://www.qub.buffalo.edu, Qin *et al*. 1996, 1997), HJCFIT (Colquhoun & Hawkes, 1994, 1995) and a MATLAB (version R2016a, MathWorks, Natick, MA, USA) implementation of the approach of Hawkes *et al*. 1990; Jalali & Hawkes, 1992; Colquhoun & Hawkes, 1995; Colquhoun *et al*. 1996, 2003). All fitting algorithms gave virtually the same results, and thus the MATLAB code was used to estimate rate constants that would maximize the likelihood that the sequence of open and closed time durations in each data record can be described by a particular model (Colquhoun & Hawkes, 1995). The MATLAB code is available from https://github.com/ogdenkev/scfit and provides functions for reading idealized transitions from SCN files created by SCAN software and DWT files created by QuB software. The code also can load channel mechanisms from Excel files or from QMF model files created by QuB software.

Separate open and shut time resolutions (also called dead times) were imposed on the data prior to fitting. Only two states were allowed for fitting, namely open and closed. Hence sojourns were considered open if the amplitude was between −0.05 and −10 pA and shut if the amplitude was less than −0.05 pA (*V*_m_, −80 mV). The first dwell time was forced to be a resolved opening (i.e. longer than the open time resolution), and the last idealized dwell was forced to be a resolved closing. The equilibrium probabilities were calculated from the Q matrix and used as the starting state probabilities, except when fitting transitions within bursts of openings defined by a critical shut time. When bursts were used for fitting, the initial and final vectors of the likelihood account for the fact that the first opening preceded a shut time greater than the critical shut time and the last shut time was at least as long as the critical shut time (see eqns (5.8) and (5.11) of Colquhoun *et al*. 1996). The exact probability density function (p.d.f.) under the assumption of missing all events shorter than the open or shut time resolution was used to calculate the likelihood for dwells up to two multiples of the dead times. For dwells longer than two multiples of the dead time, the asymptotic approximation to the exact p.d.f. was used (Hawkes *et al*. 1992; Jalali & Hawkes, 1992). The likelihood for the entire series of idealized dwells was maximized using the `fminunc' function in MATLAB. Fitting was performed on the log‐transformed transition rates, which has the effect of transforming any constraints on the rates to be linear. Any constraints on the transition rates were then formulated into a linear combination of unconstrained variables using the QR factorization method (Golub & Van Loan, 1989; Qin *et al*. 1996).

Microscopic reversibility was enforced on the rate constants in the mechanism using the minimum spanning tree method of Colquhoun *et al*. (2004). Each mechanism was treated as an undirected graph with the states equivalent to nodes and the transitions between states equivalent to edges in the graph. Then a set of rates to constrain that would enforce microscopic reversibility were found by first finding a minimum spanning tree in the graph. This was accomplished in MATLAB using the graphminspantree function. Rates in the mechanism with physical or theoretical constraints were assigned a weight in the undirected graph to ensure they would be included in the minimum spanning tree, if possible. Once the minimum spanning tree was found, graph edges not part of the minimum spanning tree were selected to be constrained. Edges corresponded to transitions between states in the gating mechanism, and because each transition is reversible there are two rates associated with each edge. Therefore, given an edge not in the minimum spanning tree, there were two choices for which rate to constrain, and one of the rates was arbitrarily selected.

For each rate selected to be constrained so that the mechanism obeys microscopic reversibility, one of the cycles in the mechanism that contained the rate was found by determining the shortest path between the pair of states connected by the rate's corresponding transition. This was done using the graphshortestpath function in MATLAB. This cycle was used to set the constraint.

After constraining rates to enforce microscopic reversibility, physical or theoretical constraints were added. All constrained rates were transformed to be linear combinations of the free rates (Colquhoun *et al*. 2004). Rate constants representing equivalent subunit conformational changes were constrained to be equal, hence reducing the number of free parameters to be estimated, while retaining the principle of describing the rates of subunit‐specific protein conformational changes. Fitting was further simplified by assuming that, at the concentrations of glutamate (1 mM) and glycine (50 μM) used for these experiments, all subunits are agonist‐bound during the recording.

### Fitting of structure‐based mechanisms to the macroscopic current responses

We also evaluated the compatibility of gating models with optimized rate constants from maximum likelihood fitting with macroscopic and synaptic current time course. We used SCALCS (http://www.ucl.ac.uk/Pharmacology/dcpr95.html) and Channelab to generate macroscopic waveforms by assuming glutamate binding and unbinding can only occur from GluN2 subunits that have not undergone a pre‐gating step. We also added desensitized states only from receptors where both subunits within a dimer had undergone pre‐gating steps (Sun *et al*. 2002; Furukawa *et al*. 2005). A model that included agonist binding was subsequently fitted simultaneously to two average macroscopic response waveforms recorded at different agonist concentrations in excised outside‐out patches under the same conditions as the single channels (e.g. pH 8.0, 0.5 mM Ca^2+^) as previously described by Erreger *et al*. (2005) using a non‐linear least squares algorithm (Channelab). We fixed the gating rates in the model to those determined from single channel maximum likelihood fitting described above and allowed the agonist association and dissociation rates in addition to rates governing entry into and exit from the desensitized states to vary (Erreger *et al*. 2005). We subsequently performed a sensitivity analysis by determining the sum of squares for normalized waveforms simulated with the agonist binding and desensitization rates varied over a range that encompassed the fitted values (see Appendix).

### Homology modelling and molecular dynamics simulations

A human GluN1/GluN2B dihetero‐tetrameric glutamate‐ and glycine‐bound receptor homology model was generated from two GluN1/GluN2B crystal/cryo‐electron microscopy structures (PDBid: 4PE5 and 5FXH; Karakas & Furukawa, 2014; Tajima *et al*. 2016). The alignment of the target and template sequences was performed in MUSCLE (Edgar, 2004). Five homology models were generated using Modeller 9v14 (Sali & Blundel, 1993) and subjected to quality analysis using the *PDBsum generator* (Laskowski, 2009). For the generation of these models, ifenprodil was excluded from being incorporated and the pocket was relaxed during a final, short molecular dynamics stage in the Modeller script as well as during the equilibration molecular dynamics run. The top scoring model had an overall G‐factor of −0.14 indicative of a high quality model, and was prepared for further analysis within Maestro using the OPLS3 force field (Schrödinger Release 2017‐1; Protein Preparation Wizard; Epik version 3.7; Impact version 7.2; Prime version 4.5, Schrödinger, LLC, New York, NY, 2017). Hydrogen bond assignments were performed followed by subsequent optimized and visual inspection. All titratable residues were assigned their dominant protonation state at pH 7.0. An energy minimization was performed on the receptor to relieve unfavourable constraints.

The molecular dynamics equilibration and production run of the system was performed using Desmond (Desmond Molecular Dynamics System, D. E. Shaw Research, New York, NY, 2017). The optimized receptor was inserted into an equilibrated palmitoyl oleoyl phosphatidyl choline (POPC) bilayer. The system was neutralized by adding 60 sodium atoms, with the salt concentration set to 150 mM (NaCl). The system consisted of 3187 amino acids, 467 lipid molecules, 82,053 waters (Simple Point Charge (SPC)) and 516 salt atoms totalling ∼360,000 atoms with a box size of 6,529,931 Å^3^. The system was relaxed using the Desmond relaxation protocol. The equilibration run was followed by a 100 ns production run performed under constant Number, Pressure and Temperature (NPT) conditions using the Berendsen thermostat (310 K and 1.103 bar) and Particle Mesh Ewald (PME) electrostatics with a cutoff of 9 Å. Time step calculations were performed every 2 fs (Δ*t*). Analysis of the molecular dynamics trajectory was performed in Desmond and Visual Molecular Dynamics (VMD) (Humphrey *et al*. 1996). The dynamic cross‐correlation of the pre‐M1 helices and protein were calculated using MD‐Task (Brown *et al*. 2017). All‐cross correlations are defined by

$$C_{ij\;}=\frac{\sum_{n}^{N}=1\frac{〈\Delta x_{i}\left(n\right)\Delta x_{j}\left(n\right)〉}{\left|\Delta x_{i}\left(n\right)\right|\times \left|\Delta x_{j}\left(n\right)\right|}}{N}$$

$$\Delta x_{i}\left(n\right)=x_{i}\left(n\right)-x_{i}\left(n-1\right)$$

where *n *= *t*/Δ*t*, *N* = number of steps (100 *ns*/Δ*t*), *t* is the simulation time, Δ*t* is the correlation interval, $x_{i}\left(n\right)$ is the position of the alpha carbon residue *i* at step *n* and *C_ij_* is the correlation between *i* and *j*.

Thus a *C_ij_* of 1 is perfect concerted movement and a *C_ij_* of −1 is perfect mirroring between residues *i* and *j* at the given time interval. Since the transmembrane domains (TMD) of adjacent subunits are positioned approximately 90° about the *y*‐axis from one another, coordinates for all four subunits throughout all frames of the simulation were rotated such that they aligned with one another in the same quadrant. This was done to avoid missing potentially correlated motions as their cross‐correlations and covariances would be calculated as zero due to orthogonality (Hünenberger *et al*. 1995). Heatmaps that illustrate completely random, uncorrelated motion of the pre‐M1 helices were generated by shuffling the sequence of coordinates for each residue in the MD trajectory by frame using the Python function ‘random.shuffle()’. Each residue's coordinates were shuffled independently from those of the other residues.

### Statistics

Data are expressed as means ± SEM and analysed using a one‐way ANOVA with Tukey's *post hoc* test. Significance for all tests was set at *P* < 0.05 and the number of observations was selected to give a power > 0.95 for a minimum detectable difference of 25% for rise time and 50% for exponential time constants (tau). Evaluation of multiple distinct parameters from the same waveform were corrected for familywise error using Holm's method of sequential adjustments. A sensitivity analysis was performed to investigate the shape of the likelihood surface around the optimum values of the rate constants estimated from single channel fitting. Statistical comparisons between models was made using a likelihood ratio test (Horn, 1987; Colquhoun & Sigworth, 1995).

## Results

### Recordings of GluN1/GluN2A single channel activity

**Figure 1:**
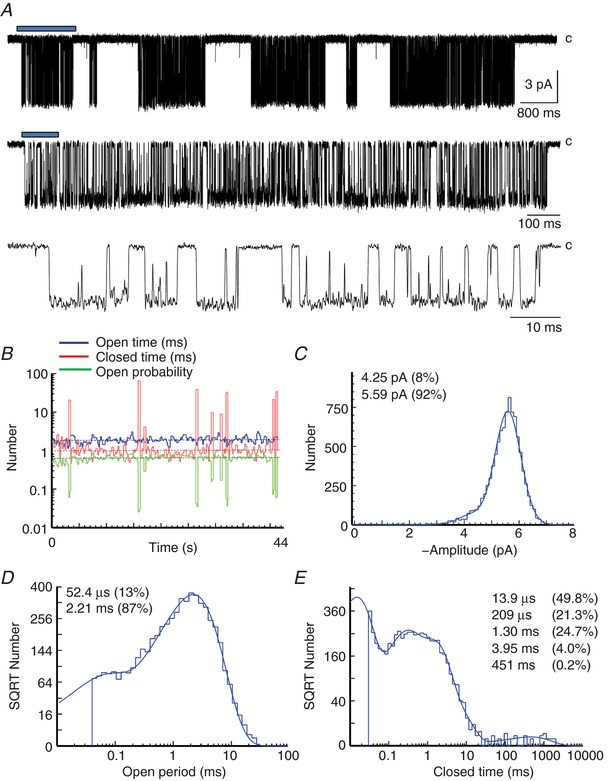
Single channel properties of GluN1/GluN2A diheteromeric NMDA receptor currents *A*, a representative recording illustrates the complex pattern of channel openings and closings observed when GluN1/GluN2A receptors are activated by high agonist concentration (1 mM glutamate, 50 μM glycine; membrane potential −80 mV). The record was filtered at 4 kHz for display. *B*, each bin in the kinetic stability plot shows a running average of 50 intervals (total 18,311 events) with increments of 25 intervals between averages. Horizontal dashed lines represent overall mean values for the whole data record (duration 44 s). Mean shut time was 2.95 ms, mean open time was 2.10 ms, and *P*_open_ was 0.416 for this patch. Resolution for openings and closings was 40 μs. *C*, the distribution of channel amplitudes was determined from time course fitting. The maximum likelihood fit of two Gaussian components (standard deviation 0.47 pA) is superimposed as a probability density function; mean amplitude and relative area are inset. *D*, distributions of channel open times were fitted with a mixture of two exponential components. *E*, distribution of channel closed times was fitted with a mixture of five exponential components.

We evaluated the properties of unitary currents in outside‐out patch clamp recordings of single rat GluN1/GluN2A receptors expressed in HEK cells. We selected a set of four outside‐out patch single channel recordings with low baseline noise that each contained one active channel for analysis. Channels were activated by saturating concentrations of glutamate (1 mM) and glycine (50 μM). We idealized these recordings at a resolution of 50 μs for open and closed times to obtain a sequence of 121,125 lifetimes as the receptor alternates between open and closed conformations of the pore (Fig. 1 *A*). We analysed recordings of individual channel openings, which were stable in duration (Fig. 1 *B*) and amplitude throughout the full experiment (Fig. 1 *C*). Analysis of the duration of open and closed lifetimes (Fig. 1 *D* and *E*) reveals the complex kinetic pattern of activity characteristic of NMDA receptor activation, as previously described in many different preparations (Jahr & Stevens, 1987; Cull‐Candy & Usowicz, 1989; Howe *et al*. 1991; Gibb & Colquhoun, 1992; Wyllie *et al*. 1998; Banke & Traynelis, 2003; Popescu & Auerbach, 2003; Auerbach & Zhou, 2005; Erreger *et al*. 2005; Erreger & Traynelis, 2008; Yuan *et al*. 2009). Table 1 summarizes the properties of these patches.

**Table 1:** Properties of GluN1/GluN2A activated with 1 mM glutamate and 50 μM glycine

Baseline r.m.s. noise	0.27 ± 0.04 pA	
Single channel current	5.41 ± 0.05 pA	
Mean open time	1.7 ± 0.2 ms	
Open probability	0.39 ± 0.06	
Open tau_FAST_	0.13 ± 0.06 ms	10 ± 2%
Open tau_SLOW_	1.8 ± 0.2 ms	90 ± 2%
Closed tau_1_	22 ± 4 μs	32 ± 2%
Closed tau_2_	0.22 ± 0.04 ms	24 ± 4%
Closed tau_3_	1.03 ± 0.09 ms	35 ± 3%
Closed tau_4_	3.3 ± 0.4 ms	9 ± 2%
Closed tau_5_	1000 ± 400 ms	0.21 ± 0.04%
Chord conductance	53 ± 2 pS	8 ± 2%
Chord conductance	69.3 ± 0.3 pS	92 ± 2%

Data are means ± SEM from 4 patches. For fitted histograms, the relative area of the individual components are given as % (right hand column). Significant figures presented were determined by the first digit of the SEM (see https://arxiv.org/ftp/arxiv/papers/1301/1301.1034.pdf). r.m.s., root mean square.

More than 90% of channel openings appeared to arise from a single conductance level, with a smaller sublevel accounting for 8% of openings. Open time histograms for all conductance levels were best described by the sum of two exponential functions, suggesting at least two energetically distinct open channel conformations (Fig. 1 *D*). For GuN2A receptors, more than 99% of closed periods were less than ∼30 ms duration. At the high agonist concentrations used in this study, the occasional long‐lived closed periods (∼1 s duration), represented by closed tau_5_ (Table 1) are much too long to represent agonist unbinding and re‐binding and so we interpret these as likely reflecting desensitized receptor conformation(s) (Fig. 1 *E*). In order to define bursts of openings, excluding the long desensitized periods, the closed time distribution exponential parameters were used to calculate the value of t‐crit (mean, 37 ± 4.8 ms). At saturating agonist concentration, closed times within bursts would also include occasions when an agonist unbinds and rebinds. However, because glutamate and glycine are high affinity agonists at the NMDA receptor, the expected frequency of agonist unbinding and re‐binding events was low (∼16 s^−1^) for the mechanisms analysed in this study, compared to the observed average of 352 channel closings per second of burst time. Thus, these closed times within bursts mainly contain information about pre‐gating receptor conformations that must be traversed en route to channel opening (Banke & Traynelis, 2003; Schorge *et al*. 2005; Erreger *et al*. 2005; Yuan *et al*. 2009). In order to fit the data with mechanisms that do not include any desensitized states, we used a t‐crit of 35 ms to identify bursts of openings, where within each burst, all openings are separated by closed periods shorter than t‐crit.

### Subunit‐dependent gating mechanism for tetrameric receptors

We first set out to use this high‐quality dataset to develop a model of the mechanism underlying GluN1/GluN2A NMDA receptor activation that would reflect structural features of receptor function. Our goal was to relate the activation steps that follow ligand binding to conformational changes in individual subunits that would allow subsequent analysis of receptors with non‐identical GluN1 or GluN2 subunits. We therefore focused on modelling the resolvable, kinetically distinct conformations (out of many that must exist) necessary to describe the behaviour of a fully liganded receptor prior to agonist‐induced channel opening. Because the receptor is a tetrameric protein containing two GluN1 and two GluN2 subunits, we describe a mechanism that allows a pre‐gating conformational change that is influenced by each of the four subunits to occur in any order. Each of the receptor protein conformational changes inferred from the data record could reside entirely within a single subunit, or reflect unique interactions between subunits (Ogden *et al*. 2017; Chen *et al*. 2017). We envision these pre‐gating changes to involve one or more of the short linker segments connecting the bi‐lobed agonist binding domains to the transmembrane elements, as perturbations in the linkers can perturb receptor function (Talukder & Wollmuth, 2011; Kazi *et al*. 2013, 2014; Ogden *et al*. 2017) while recognizing that, in principle, changes in any part of the protein structure may affect function. Because the linkers exist at the interface between two‐ and fourfold symmetry, we speculate these movements are likely distinct for GluN1 and GluN2 subunits as suggested by comparison with the analogous AMPA receptor GluA2 structure (Twomey *et al*. 2017).

**Figure 2:**
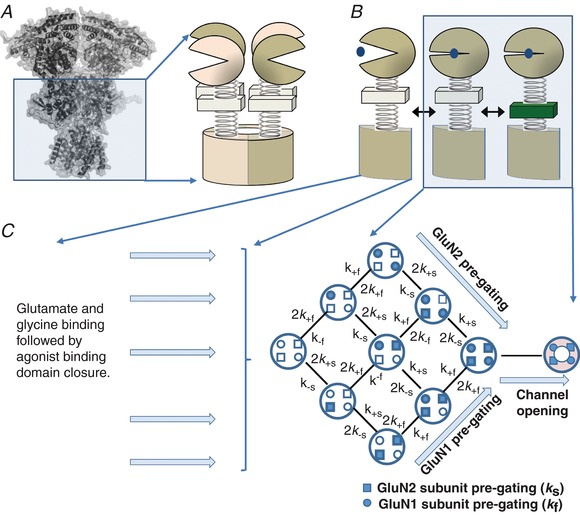
A model of subunit‐dependent conformational changes for a tetrameric diheteromeric receptor *A*, space‐fill of the GluN1/GluN2B receptor is shown (Karakas & Furukawa, 2014; Lee *et al*. 2014) with the ligand binding domain and pore shown as a diagrammatic representation of the tetrameric assembly of subunits. *B*, illustration of steps within each subunit hypothesized to precede opening of the pore. These include the binding of agonist to the bi‐lobed agonist binding domain that involves domain closure (left) and a conformational change in a linker between the agonist binding domain and the transmembrane domain that is distinct from domain closure (right). *C*, a mechanism is shown that treats each subunit as independently able to undergo a pre‐gating conformational change once the ligand binding sites are occupied. This allows subunits to participate in a pre‐gating conformational change in any order, and when all four subunits have undergone this change, the probability that the ion channel rapidly opens in an all‐or‐none fashion is greatly increased. Ligand binding steps are not represented in this mechanism.

Figure 2 illustrates this mechanism, which conceptualizes some of the key steps in the response of a synaptic receptor to glutamate release that we seek to evaluate. For simplicity we hypothesize that following agonist binding, each subunit undergoes some as yet unknown conformational change (that will also involve residues of adjacent subunits) in a key gating region, represented in the diagram in Fig. 2 *A* and *B* as a change in the position of a spring connecting the ligand binding domain to the channel. When this change has occurred in some subset of the four agonist‐bound subunits that comprise the tetrameric receptor, the probability that the pore will rapidly dilate to allow current flow is greatly increased. Figure 2 *C* shows one hypothetical example of this in which opening occurs when all four subunits undergo this pre‐gating step. However, a single open state is not sufficient to account for the data. Two different open states are needed to account for the consistent presence of two exponential components in distributions of single channel open times in our data set (Fig. 1 *D*) and across many GluN2A receptor studies (Qin *et al*. 1996; Wyllie *et al*. 1998; Popescu *et al*. 2004; Erreger *et al*. 2005; Schorge *et al*. 2005; Auerbach & Zhou, 2005). The model in Fig. 2 contains nine channel closed states, which are sufficient to reflect the most complex closed time histograms described in the literature reporting five or six distinguishable exponential components (Fig. 1 *E*) (Wyllie *et al*. 1998, 2006; Popescu *et al*. 2004; Erreger *et al*. 2005; Auerbach & Zhou, 2005; Yuan *et al*. 2009). Imposition of constraints based on the symmetry of the tetramer, the assumption that the rate of a conformational change in a subunit is constant regardless of changes in other subunits (see also dimer model below) and enforcement of microscopic reversibility together reduce the number of free parameters considerably.

### Pre‐M1 helix controls the rate of channel activation after agonist binding

We and others have previously evaluated the functional effects on channel properties of disease‐associated *de novo* mutations located in or near the pre‐M1 helix (e.g. Alsaloum *et al*. 2016; Ogden *et al*. 2017). This two‐turn cuff helix lies parallel to the outer leaf of the plasma membrane and is in van der Waals contact with the M3 transmembrane helix of the same subunit, which is thought to form the channel gate (Sobolevsky *et al*. 2009; Karakas & Furukawa, 2014, Lee *et al*. 2014; Twomey & Sobolevsky, 2017). In addition, the GluN2 pre‐M1 helix is in close proximity to conserved residues in the pre‐M4 linker shown to critically control gating (Talukder *et al*. 2010; Yuan *et al*. 2014; Amin *et al*. 2017; Chen *et al*. 2017). We have proposed that this trio of interactions (GluN1‐pre‐M1/GluN1‐M3/GluN2‐pre‐M4 and the GluN2‐pre‐M1/GluN2‐M3/GluN1‐pre‐M4) are involved in the stabilization of the closed state, for which a helical bundle crossing of all four M3 helices occludes ion permeation (Ogden *et al*. 2017; Chen *et al*. 2017). We further hypothesize that conformational changes in pre‐M1/M3/M4 interactions in at least a subset of subunits is a prerequisite to the rapid, coordinated movement of the M3 helices that results in an almost instantaneous opening of the channel, giving the unitary current flowing through one channel its square step‐like appearance. We suggest that these two unique gating triads present in each GluN1/GluN2 dimer each undergo the required pre‐gating conformational changes at distinguishable rates, and thus are candidates for the physical basis of pre‐gating steps evident in shut time histograms from agonist‐bound receptors (Banke & Traynelis, 2003; Popescu & Auerbach, 2003; Schorge *et al*. 2005; Erreger *et al*. 2005). A role for the M3 helices generating channel opening is consistent with the large number of disease‐associated variants located in these regions that affect channel open probability (e.g. Yuan *et al*. 2014; Ogden *et al*. 2017; Chen *et al*. 2017).

**Figure 3:**
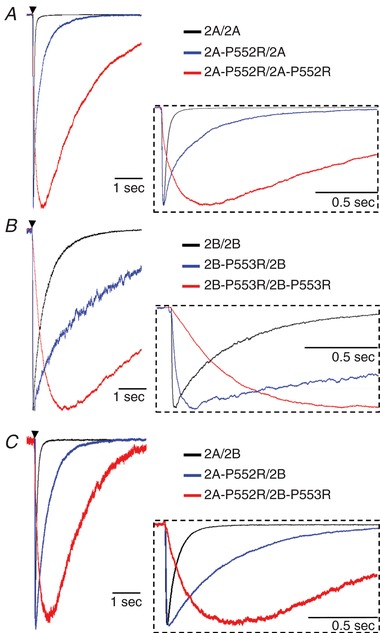
Representative current responses following brief (5 ms, black arrow) application of glutamate (1000 μM) plus glycine (30 μM) onto cells bathed in glycine GluN1/GluN2A receptors (*A*) and GluN1/GluN2B receptors (*B*) containing zero (black), one (blue), or two (red) mutated GluN2 subunits. A single copy of the mutation slows deactivation by 6‐fold for GluN1/GluN2A and 5‐fold for GluN1/GluN2B. Two copies of the mutation slows deactivation by 46‐fold for GluN1/GluN2A and 11‐fold for GluN1/GluN2B. *C*, representative traces following the application of glutamate (1000 μM) plus glycine (30 μM) for 5 ms (black arrow) onto cells bathed in glycine (30 μM) and expressing GluN1/GluN2A/GluN2B (black), GluN1/GluN2A‐P552R/GluN2B (blue), or GluN1/GluN2A‐P552R/GluN2B‐P553R (red). GluN1/GluN2A‐P552R/GluN2B and GluN1/GluN2A‐P552R/GluN2B‐P553R slow deactivation by 7‐fold and 24‐fold, respectively. For all panels, the inset shows that a single copy of the mutation does not alter the response rise time, but two copies of the mutation slows the rise time.

The *de novo* disease‐associated mutation P552R in the pre‐M1 GluN2A (but not GluN1 pre‐M1) alters NMDA current rise time in response to rapid glutamate application in a manner dependent on the number of mutant GluN2A subunits present in the receptor (Ogden *et al*. 2017). This suggests that each subunit may independently undergo a distinct conformational change that precedes gating (Fig. 2). To explore this point further, we expressed GluN1/GluN2A receptors that contained 0, 1 or 2 copies of GluN2A‐P552R. To determine if this was a general phenomenon, we also expressed GluN1/GluN2B with 0, 1 or 2 copies of the analogous mutation (P553R). After applying glutamate to each cell for a brief duration (5 ms), we rapidly moved each cell to a glutamate‐free solution to evaluate the current response rise time in the absence of glutamate. We reasoned that under this protocol the rate of rise of the current response (estimated by least‐squares fit to the currents as a function of time) must reflect conformational changes after agonist binding and prior to channel opening, since glutamate was removed from the extracellular solution after a few milliseconds, well before the receptor had fully activated. We found that receptors with 0 or 1 copy of GluN2A‐P552R or GluN2B‐P553R can become activated at rates similar to wild type receptors, as there was no detectable difference between the 10–90% current response rise times (Table 2). However, receptors with two copies of GluN2A‐P552R or two copies of GluN2B‐P553R respond over an order of magnitude more slowly, with the response continuing to rise long after glutamate had been removed by the rapid perfusion system (Fig. 3 *A* and *B*). The lack of effect on activation rate of one mutated GluN2 subunit in the tetrameric receptor complex supports the idea that NMDA receptor open states can be reached when either one or both GluN2 subunits have undergone some conformational change. The ability of a channel to open at the same rate as wild type even with one impaired GluN2 subunit suggests that the receptor may reach an open state independent of the conformation of the second, mutant GluN2 subunit.

**Table 2:** Di‐ and triheteromeric NMDA receptor macroscopic response properties

	Rise time (ms)	tau_1_ (ms)	tau_2_ (ms)	% tau_1_	Weighted tau (ms)	*n*
2A/2A	8.3 ± 0.5	33 ± 3.0	147 ± 28	88 ± 3.5	41 ± 3.7	12
2A‐P552R/2A	9.5 ± 1.0	67 ± 16	367 ± 38	49 ± 8.1	260 ± 39	9
2A‐P552R/2A‐P552R	230 ± 14*†	1200 ± 140	4040 ± 655	77 ± 19	1900 ± 180*†	8
2B/2B	15 ± 1.0	210 ± 16	744 ± 31	46 ± 13	496 ± 25	16
2B‐P553R/2B	43 ± 3.2	310 ± 29	3990 ± 255	37 ± 4.0	2600 ± 140*	8
2B‐P553R/2B‐P553R	960 ± 83*†	5780 ± 300	—	100 ± 0	5780 ± 300*†	8
2A/2B	8.0 ± 0.3	64 ± 6.6	425 ± 101	91 ± 2.0	78 ± 5.7	14
2A‐P552R/2B	12 ± 1.0	440 ± 76	1790 ± 394	84 ± 8.7	540 ± 58*	9
2A‐P552R/2B‐P553R	330 ± 21*†	1900 ± 130	—	100 ± 0	1900 ± 130*†	9

NMDARs were expressed in HEK cells, and whole cell current recordings made under voltage clamp in response to the rapid application of glutamate for 5 ms (see Methods). Means ± SEM are shown for all parameters to two significant figures where '*n*' is the number of observations. The weighted tau was determined from the fitted tau values from two exponential functions, with the exception of 2B‐P553R/2B‐P553R and 2A‐P552R/2B‐P553R, which deactivated with a time course described by only one exponential. ^*^ *P* < 0.001 compared to wild type, one‐way ANOVA, Tukey *post hoc*, multiple comparisons, ^†^ *P* < 0.001 compared to single‐copy mutants, one‐way ANOVA, Tukey *post hoc*, multiple comparisons. *F* values for rise time for 2A/2A, 2B/2B and 2A/2B were *F*_2,26_ = 347, *F*_2,29_ = 198 and *F*_2,30_ = 295, respectively. *F* values for weighted tau for 2A/2A, 2B/2B and 2A/2B were *F*_2,26_ = 118, *F*_2,29_ = 235 and *F*_2,30_ = 161, respectively. The sequential approach of Holm was used to correct familywise error.

### Triheteromeric receptors may open after a single GluN2 subunit has undergone pre‐gating

A large number of NMDARs in the central nervous system contain two different GluN2 subunits, including hippocampal synaptic receptors that contain GluN1/GluN2A/GluN2B (Rauner & Köhr, 2011; Tovar *et al*. 2013). To evaluate whether these triheteromeric receptors can activate normally when one GluN2 subunit contains a function impairing pre‐M1 Pro–Arg substitution, we co‐expressed GluN1/GluN2A/GluN2B triheteromeric receptors with an engineered C‐terminal that allowed for control over the stoichiometry of cell surface receptors (Hansen *et al*. 2014). We introduced a single copy of GluN2A‐P552R (Ogden *et al*. 2017) into the triheteromeric receptor complex and assessed the response time course (Fig. 3 *C*). We used the GluN2B‐selective inhibitor ifenprodil (Hansen *et al*. 2014) in control experiments to ensure that the currents we recorded reflected triheteromeric receptors. For cells transfected with GluN1/GluN2A_C1_‐P552R/GluN2B_C2_, if appreciable GluN2B_C2_ escaped the ER and formed diheteromeric GluN1/GluN2B_C2_ receptors that reached the surface, then the observed peak current will be higher than that of the triheteromeric receptor alone.

The GluN2B selective inhibitor ifenprodil at 3 μM should reduce the peak current of diheteromeric GluN1/GluN2B_C2_ receptors by ∼90%, while reducing the triheteromeric peak current by ∼30%. We observed only partial block (38%) of the current amplitude of responses from GluN1/GluN2A_C1_/GluN2B_C2_ transfected cells, consistent with the previously reported effects of ifenprodil on GluN2A/GluN2B triheteromeric receptors (Hatton & Paoletti, 2005; Hansen *et al*. 2014). Additionally, when applied to GluN2A_C1_‐P552R/GluN2B_C2_ and GluN2A_C1_‐P552R/GluN2B_C2_‐P553R triheteromeric receptors, 3 μM ifenprodil reduced peak current by 58 ± 5.5% (*n* = 9) and 43 ± 6.1% (*n* = 9), respectively. The effects of ifenprodil on current responses from cells transfected with GluN1, GluN2AC1‐P552R and GluN2B_C2_ suggest that contributions to macroscopic current by diheteromeric GluN1/GluN2B_C2_ receptors that escaped ER retention are minimal.

As observed with diheteromeric receptors (Fig. 3 *A* and *B*), introduction of a single copy of GluN2A‐P552R mutation in pre‐M1 did not markedly slow the current response rise time of triheteromeric GluN1/GluN2A‐P552R/GluN2B receptors to brief (5 ms) application of glutamate (Fig. 3 *C*; Table 2), suggesting a triheteromeric GluN2A/GluN2B receptor can open when a single wild type GluN2B subunit has undergone pre‐gating. By contrast, triheteromeric receptors where both GluN2 subunits carry the mutation, 2A‐P552R/2B‐P553R activate approximately 30‐fold more slowly (Table 2). Because the functional effect of the GluN2 proline mutation is conserved between 2A and 2B and across the 2A/2B triheteromer, this result strongly supports the conclusion that the pre‐M1 region of GluN2A and 2B subunits is an important determinant of the rate of receptor activation.

### Pre‐M1 helices move independently between subunits in molecular dynamics simulations

**Figure 4:**
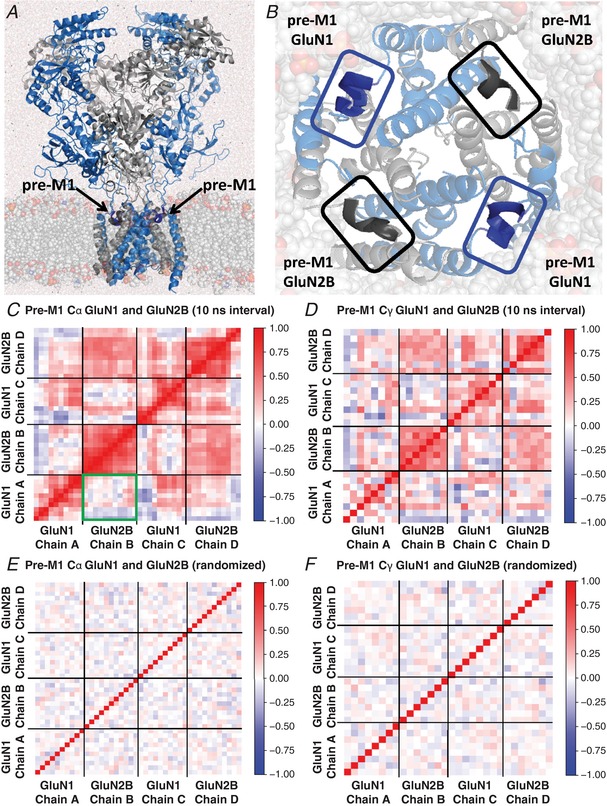
Simulated movement of pre‐M1 helices is not strongly correlated across subunits *A*, the hydrated GluN1/GluN2B (blue/grey) receptor was embedded in a lipid bilayer. *B*, the pre‐M1 linker regions are expanded with the transmembrane region and the membrane (translucent). *C*, heatmap of the dynamic cross‐correlation between the Cα atoms from each of 10 residues in the pre‐M1 of GluN1 (residues 551–560) and 11 residues in the pre‐M1 of GluN2B (residues 547–557) for chains A–D (chains labelled clockwise from top left) was determined from the first 500 ns of a 1 μs molecular dynamics run; identical results were obtained when analysing the full run (not shown). The section in the green box highlights a lack of correlation between pre‐M1 helices of adjacent subunits. *D*, the cross‐correlation of the Cγ atoms for 8 residues from the pre‐M1 of GluN1 and 7 residues from the pre‐M1 of GluN2B for chains A–D is shown. The red boxes forming a diagonal line indicate a perfect correlation of either the Cα or Cγ atoms of the pre‐M1 for both the GluN1 and the GluN2B chains to themselves. Only weak inverse correlation is observed with the correlation coefficient *r* ranging between 0 and 0.57; weak inverse correlations for subunits on opposite sides of the receptor are shown as red due to the rotation of the subunits to overcome orthogonality to the adjacent chains as described in the Methods. The cross‐correlation measurements shown at time intervals of 10 ns throughout the 500 ns simulation were consistent with those in the other time intervals. *E* and *F*, heatmaps were generated by shuffling the frames from the molecular dynamics trajectories shown in *C* and *D* to depict the absence of correlation that would be observed for completely random motion of the helices using 10 ns intervals for each Cα and Cγ, respectively. Cross correlations were determined as described in the Methods.

An assumption of the tetrameric models is that the pre‐gating steps can occur independently in different subunits. While the flexible nature of the pre‐M1 and pre‐M4 linkers is consistent with this idea, the close association of these two elements with the SYTANLAAF region of the M3 helices, which themselves are in close association with the M3 regions on other subunits, raises the possibility that the movement of the pre‐gating linker regions are correlated between subunits, and not fully independent. To test this idea, we ran a 1000 ns molecular dynamics simulation on a full‐length solvated GluN1/GluN2B dimeric receptor embedded in a lipid bilayer (Fig. 4 *A* and *B*). We chose GluN1/GluN2B for molecular dynamics simulations because a crystallographic dataset exists for this receptor (see Methods), and because of data (see Fig. 3) showing similar dependence of GluN1/GluN2A and GluN1/GluN2B macroscopic rise time on the number of mutant GluN2 subunits in the complex. The structures used as our starting points were crystallized in the presence of glutamate, glycine and ifenprodil (Karakas & Furukawa, 2014) and so are in an ‘active but inhibited’ conformation. Ifenprodil was excluded from the generation of the glutamate‐ and glycine‐bound model that we ran molecular dynamics on (Tajima *et al*. 2016). Therefore, we hypothesize that in this structure, pre‐gating motions are possible. We evaluated the cross‐correlation of the pre‐M1 helix of GluN1 and GluN2B at 100, 500, 1000, 5000 and 10,000 ps intervals.

The analysis was conducted with respect to a trajectory aligned on the four M3 helices of diheteromeric GluN1/GluN2B. This alignment ensures that the correlations measured reflect movement of the residues in the pre‐M1 helix and eliminated noise introduced by rigid body motion of the receptor as well as local domain shifting when correlations are calculated based on the full receptor. Red pixels in the correlation maps in Fig. 4 *C* and *D* signify concerted movement in the same direction of Cα atoms (Fig. 4 *C*) and Cγ atoms (Fig. 4 *D*) of amino acid residues 551–560 from GluN1 (LDSFMQPFQS) and 547–556 of GluN2B (PSAFLEPFSA) while the blue regions represent fluctuations, which are negatively correlated for subunits when geometrically superimposed. The heatmaps show the lack of correlation of movement of the Cα and Cγ atoms of the pre‐M1 helices (Fig. 4 *C–F*). At steps of up to 10 ns we found only weak inverse correlation of the pre‐M1 regions, consistent with the assumption of independence of these linker regions (Fig. 4 *C*). Although, in principle, the subunits of a multi‐subunit protein are never entirely independent, these analyses suggest GluN2 pre‐M1 helices move independently in closed channels, supporting representation of these steps as independent in our gating scheme.

### Explicit subunit pre‐gating steps can describe GluN1/GluN2 single channels

**Figure 5:**
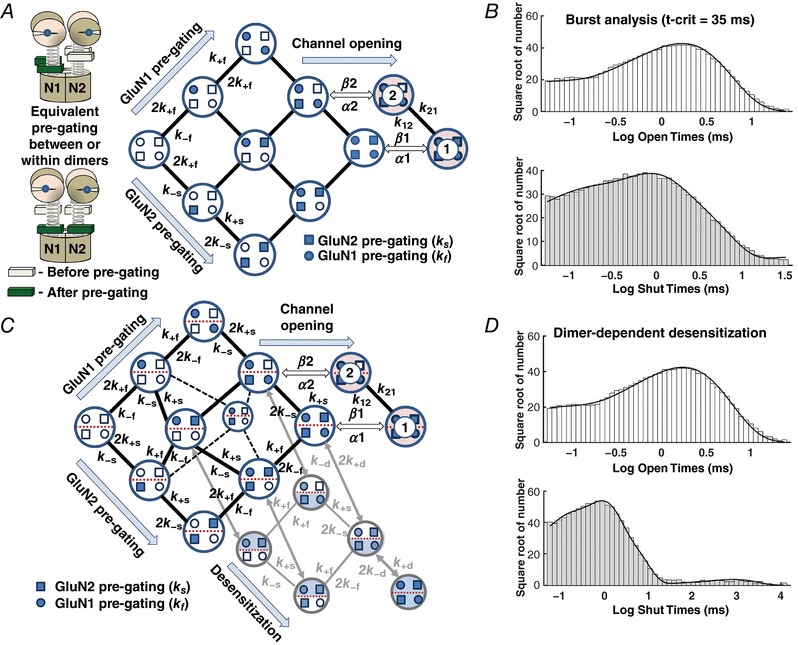
Fitting a tetrameric NMDAR model to single channel GluN1/GluN2A data *A*, pre‐gating conformational changes within each subunit are explicitly shown for all four subunits. Rate constants subscripted ‘f’ refer to GluN1 subunit‐dependent transitions and ‘s’ refer to GluN2 subunit transitions. Greek letters β and α refer to channel opening and closing rates, respectively, while the transition rates between open states 1 and 2 are denoted *k*_12_ and *k*_21_. For clarity, not all rate constants are shown. Inset cartoon illustrates the fourfold symmetry of the transmembrane region and no interaction between subunits within or between dimers at the level of the pre‐gating conformational change. *B*, the best fit of this mechanism to all data simultaneously (including correction for limited recording bandwidth; see Methods) produces predicted open and closed time distributions that closely match those recorded, as shown by the superposition of predicted and measured closed and open time distributions. *C*, a mechanism that explicitly considers the pre‐gating transitions within subunit dimers (delineated by dashed red lines) and dimer‐dependent desensitization (with rate constants *k*_+d_ and *k*_‐d_). Desensitization may occur whenever both subunits in a dimer have undergone pre‐gating transitions. Note that for a receptor where one GluN1 and one GluN2 subunit have undergone pre‐gating transitions, either the subunits are in the same dimer, or they are in separate dimers. *D*, the best fit of this mechanism to all data (without use of t‐crit) produces open and closed time distributions that closely match those recorded, as shown by the superposition of predicted and measured closed and open durations shown as histograms.

We subsequently developed an explicit activation mechanism, illustrated in Fig. 5, for a receptor that contains two identical GluN1 and two identical GluN2 subunits, with two open states. Based on this mechanism, each agonist‐bound subunit of the receptor can independently undergo a pre‐gating conformational change, perhaps in the pre‐M1 helix (indicated by open or filled symbols) in the GluN1 subunits (rates *k*_+f_ and *k*_ −f_) or GluN2 subunits (rates *k*_+s_ and *k*_−s_) in any order (Fig. 5 *A*). The two open states can interconvert (rate constants *k*_12_ and *k*_21_), and are reached by distinct opening rates, β_1_ and β_2_ (α_1_ and α_2_ are distinct closing rates). The model is constructed assuming the glycine and glutamate binding sites on the GluN1 and GluN2 subunits, respectively, are occupied at high concentrations of glycine (50 μM, > 35 × EC_50_) and glutamate (1 mM, ∼300 × EC_50_) relative to their EC_50_ values. We assumed that there is no cooperativity in these conformational changes nor in the dimer‐dependent desensitization rates, such that subunit transition rates are constant regardless of the conformational state of the other three subunits or whether changes occur within the partner dimer. Consideration of results with one mutant and one wild type GluN2 subunit (Fig. 3) led to the hypothesized open state connectivity where channel opening may occur when both GluN1 subunits and either one (or both) of the GluN2 subunits has undergone pre‐gating.

The data shown above (Fig. 3) for the human *de novo* mutation GluN2A‐P552R and the analogous mutation GluN2B‐P553R suggest a possible explanation as to why a receptor with two mutant GluN2A subunits opens ∼30‐fold more slowly than wild type receptors, while a receptor with a single mutant subunit can open at a rate comparable to wild type receptors. Receptors engineered to contain only one of these mutant subunits activated with a rate very similar to wild type receptors, raising the possibility that only one GluN2 subunit needs to undergo pre‐gating before the channel can open. Thus GluN1‐GluN2AP552R‐GluN2B receptors had a 10–90% rise time (12 ms) that is reminiscent of a diheteromeric GluN1‐GluN2B receptor (15 ms), as opposed to GluN1‐GluN2A (8 ms). We predict that the converse construct of GluN1‐GluN2A‐GluN2BP553R would have a 10–90% rise time that is close to GluN1‐GluN2A. However, we could not confirm this in these experiments as a pharmacological tool equivalent to ifenprodil that would allow a significant contribution of GluN2A diheteromers to the macroscopic current to be discounted is not available for GluN2A receptors. Compared to the mechanism described in Fig. 2, the mechanism shown in Fig. 5 *A* accounts for the idea that channel opening may occur after only one of the GluN2 subunits have undergone pre‐gating by allowing a second conformation (two GluN1 active, one GluN2 active) within the scheme to undergo channel opening. Having two open states is consistent with the presence of two exponential components in the channel open time distribution (Fig. 1, Table 1), and the idea that opening can occur when not all subunits are active is reminiscent of AMPA receptors, which can open without agonist binding to all four subunits (Rosenmund *et al*. 1998).

Several studies have found significant negative correlation between the duration of an opening and the duration of the adjacent shut time for NMDA receptors (Gibb & Colquhoun, 1992; Schorge *et al*. 2005; Wyllie *et al*. 2006). Correlations are evident in conditional distributions of open times of hippocampal NMDA receptors, but are not strong for diheteromeric GluN2A receptors (Auerbach & Zhuo, 2005; Erreger *et al*. 2005). This data set had a small but statistically significant negative correlation between the duration of adjacent open and shut times (*r* = −0.015; *P* < 0.01) indicating that there is more than one ‘gateway’ state between open and closed states (Colquhoun & Hawkes, 1987), which is accounted for in the model shown in Fig. 5 *A* by allowing receptors with two GluN1 and one GluN2 active subunits to open. The correlations in the reaction scheme are implicitly taken into account in our implementation of maximum likelihood fitting of the mechanism to the sequence of open and closed times in the data record (Hawkes *et al*. 1992). Maximum likelihood fitting of this model to the sequence of open and closed durations within bursts identified with a t‐crit of 35 ms (see Methods) revealed that this mechanism fits the data from the four GluN1/GluN2A patches analysed here remarkably well (Fig. 5 *B*). Table 3 summarizes the rates obtained from fitting the model in Fig. 5.

**Table 3:** Summary of model fitted rate constants

	Non‐desensitizing model, burst analysis (t‐crit 35 ms)			Desensitizing model	
	Composite fit (s^−1^)	Mean ± SEM (s^−1^)		Composite fit (s^−1^)	Mean ± SEM (s^−1^)
*k*_12_	568	1170 ± 656	*k*_12_	1390	2330 ± 906
*k*_21_	17700	16400 ± 2270	*k*_21_	15200	12900 ± 2560
α_1_	525	428 ± 87	α_1_	344	250 ± 71
β_1_	4580	4390 ± 1190	β_1_	4130	3300 ± 919
α_2_	8730	8080 ± 818	α_2_	5670	4830 ± 1290
β_2_	1580	2350 ± 944	β_2_	3550	4840 ± 1190
*k*_+f_	5000	4940 ± 560	*k_+_*_f_	4580	5470 ± 854
*k*_−f_	2500	2630 ± 301	*k*_−f_	2500	3420 ± 818
*k*_+s_	11.1	14.0 ± 5.17	*k*_+s_	256	236 ± 19
*k*_−s_	8.56	9.31 ± 1.60	*k*_−s_	225	230 ± 47
*k*_+d_	—	—	*k*_+d_	5.28	4.57 ± 0.860
*k*_−d_	—	—	*k*_−d_	1.78	2.34 ± 0.470
LL	−41,728	−41,403	LL	−42,130	−41,805

Rate constants estimated from composite fit of all patch data (first column) and given as the mean ± SEM to 3 significant figures from fits to each of 4 patches. The total number of open–closed transitions was 108,467; LL is the log‐likelihood.

### Dimer‐dependent receptor activation and desensitization

While the burst analysis presented above provides an excellent fit to the data, we were surprised by the unusually slow rate constants representing GluN2 subunit pre‐gating (*k*_+s_ rates ∼10 s^−1^; Table 2). These slowed the simulated macroscopic activation to a rate incompatible with the kinetics of GluN2A receptor relaxations (Wyllie *et al*. 1998; Vicini *et al*. 1998; Popescu & Auerbach, 2003; Popescu *et al*. 2004; Erreger *et al*. 2005). We reasoned that these slow rates may partly reflect optimization of the model to account for the proportion of the sojourns into desensitized states that are of shorter duration than t‐crit of 35 ms. To test whether such misclassification of closed times following isolation of bursts with a t‐crit impacted the determination of fitted rates for subunit pre‐gating steps, we developed a second model to fit to the full data set (without application of a t‐crit) in which the receptor can undergo a desensitizing conformational change. Because glutamate receptor desensitization is a dimer‐dependent phenomenon (Sun *et al*. 2002) and NMDA receptors have a dimer‐of‐dimers structure for the agonist binding domains (Furukawa *et al*. 2005), we modified the model shown in Fig. 5 *A* to include recognition of when GluN1 and GluN2 pre‐gating steps have occurred within a dimer (Alsaloum *et al*. 2016). We further assume that desensitization is possible whenever one GluN1 and one GluN2 subunit within the same dimer have undergone a pre‐gating conformational change. We therefore termed this a ‘dimer‐dependent desensitization’ model in order to capture the extra connectivity in this model. Figure 5 *C* illustrates this concept. An advantage of using this approach is that all information in the data record are included in model fitting without exclusion of long closed periods. In addition, this dimer mechanism includes two explicit configurations for when one GluN1 and one GluN2 pre‐gating step has occurred: either both occur within a dimer, or each occurs in separate dimers. Figure 5 *D* illustrates that maximum likelihood fitting of this mechanism to the single channel data provides an excellent description of the data. Table 3 summarizes the rate constants found from fitting these data with the dimer‐dependent desensitization model. The predicted histograms reveal a modest improvement in the fit, which can be observed in slightly higher maximum log likelihood (−41,728 *vs*. −41,730, see Table 3). Thus, explicit representation of a dimer‐dependent conformational change is compatible with the data for diheteromeric GluN1/GuN2A receptors. Interestingly, inclusion of desensitized states in the model accelerated the estimated rate constants for the hypothesized GluN2 subunit steps (*k*_s_ rates > 100 s^−1^), consistent with the idea that even a small number of closed periods in the single channel record misclassified as within bursts can impact on determination of the model parameters (Colquhoun *et al*. 2003; Schorge *et al*. 2005).

### The tetrameric subunit‐dependent mechanism predicts synaptic response properties

One goal in this exploration of a structurally based NMDA receptor activation mechanism is to gain insight into synaptic function of NMDA receptors in health and disease. Thus, a crucial test of new mechanisms with rates derived from fitting single channel data is that they should also reproduce the properties of synaptic receptors. For GluN1/GluN2A receptors, the model should respond to a rapid synaptic‐like pulse of 1 mM glutamate with a rapid rise time and high peak *P*_open_ (e.g. *P*_open_ ∼0.5, 10–90% rise time of < 10 ms). The deactivation time course following rapid removal of glutamate, as occurs at the synapse, should be compatible with both the synaptic exponential time course, with a weighted tau close to time constants (50–90 ms) reported for GluN2A‐containing synaptic receptors in cerebellar neurons (Traynelis *et al*. 1993; Cathala *et al*. 2000; Prybylowski *et al*. 2002; Lu *et al*. 2006), as well as the deactivation time course for recombinant GluN1/GluN2A receptors (weighted tau ∼40 ms).

**Figure 6:**
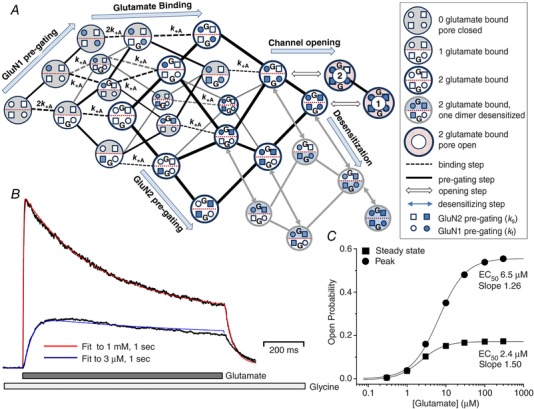
Glutamate binding to a structurally based model of receptor activation *A*, glutamate binds prior to subunit‐dependent pre‐gating steps (glycine is assumed to be bound at all subunits). For each binding step (dashed lines), only the association rate *k*_+A_ is shown for clarity; the dissociation rate *k*_‐A_ is not shown. *B*, macroscopic currents from GluN1/GluN2A were recorded from excised outside‐out patches in response to rapid application of 1 mM or 3 μM glutamate for 1 s. A least squares fitting algorithm was used to optimize glutamate association and dissociation rates and the rates describing the entry and exit from the desensitized state by simultaneously fitting the model in *A* to both waveforms. The best fit is shown superimposed onto the macroscopic currents. *C*, simulated concentration–effect curves for the peak and steady state response to a 2 s application of variable concentrations of glutamate. The smooth curves are the Hill equation fitted to the simulated data according to Response = Maximum/(1 + (EC_50_/[glutamate])${}^{N_{H}}$) where EC_50_ is the concentration of glutamate that generates a half‐maximal response and *N*_H_ is the Hill slope.

Figure 6 *A* shows our conceptualization of the binding of glutamate to the tetrameric model shown in Fig. 5 *A*. We assume a reciprocal relationship between the pre‐gating step and agonist binding, where by analogy with nicotinic acetylcholine receptors (Auerbach, 2013), the probability of the subunit gating step transition is very low (but not zero) for the unbound subunit and relatively high for the bound subunit. We also assume that once the subunit pre‐gating step has occurred (and also if a dimer has desensitized), the probability of agonist unbinding is very low. Similarly, we assume glutamate only binds appreciably to GluN2 subunits for which the pre‐gating step has not occurred. We do not represent glycine binding, and assume in this model that the GluN1 subunits have bound glycine at all times. It may be that pre‐gating steps require both glutamate and glycine to be bound in a dimer, although this is not tested here. We also explicitly model desensitization as a dimer‐dependent phenomenon, with desensitization only occurring from receptor conformations where all four subunits have bound agonist and for which both subunits within a heterodimer are activated (Furukawa *et al*. 2005). Two further desensitizing steps are possible in this model if desensitization of one dimer can occur while the GluN2 subunit of the partner dimer has not bound glutamate. However, addition of these desensitizing steps did not appreciably alter the macroscopic response characteristics of the receptor or rates inferred from fitting single channel data (data not shown) and so these are not included in Fig. 6 *A*.

Figure 6 *B* shows the average macroscopic current responses of GluN1/GluN2A receptors in excised patches (*n* = 9) activated by 1 s pulses of 1 mM or 3 μM glutamate co‐applied with saturating glycine (100 μM), which was also in the control solution. The response characteristics are consistent with those previously described for recombinant diheteromeric GluN1/GluN2A receptors, and show the expected activation time course: pronounced desensitization in the sustained presence of agonist and rapid deactivation following glutamate removal (Wyllie *et al*. 1998; Vicini *et al*. 1998; Popescu *et al*. 2004; Erreger *et al*. 2005). To determine the microscopic glutamate association and dissociation rates, we fitted the model shown in Fig. 6 *A* simultaneously to the two waveforms with pre‐gating rates fixed to those determined from fitting single channel data (Table 3), as previously described (Erreger *et al*. 2005). This approach allows estimation of the glutamate association and dissociation rates, as well as the desensitization rates that can best reproduce the slow, complex rise time observed for submaximal agonist concentrations as well as the rapid activation and slower deactivation observed when NMDA receptors are briefly exposed to a saturating concentration of glutamate. From this least squares fitting we obtained a microscopic glutamate association rate *k*_+A_ of 6.7 × 10^6^ M^−1^ s^−1^ and a dissociation rate *k*_‐A_ of 65 s^−1^. We also determined from these macroscopic responses the rates for the onset of (*k*_+d_ = 7.41 s^−1^) and recovery from desensitization (*k*_−d_ = 1.54 s^−1^). The desensitization rates were nearly identical to those determined from fitting the single channel records (*k*_+d_ = 5.28 s^−1^, *k*_−d_ = 1.78 s^−1^, Table 3). While fitted rates are model dependent because the connectivity of states differs between models, the fitted glutamate association and dissociation rates here are nevertheless remarkably similar to the rates previously described for GluN1/GluN2A receptor agonist binding (Popescu *et al*. 2004; Schorge *et al*. 2005).

**Figure 7:**
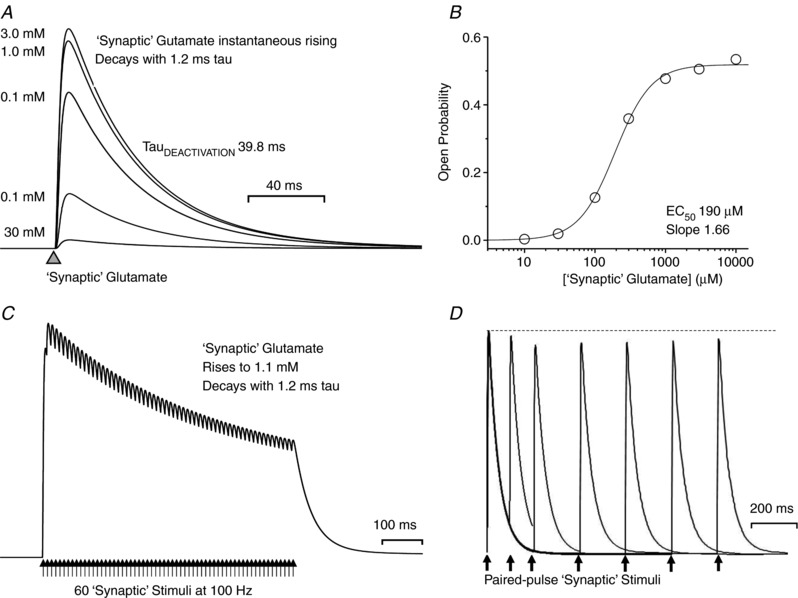
Fitted parameters predict synaptic signalling characteristics *A*, simulated responses are superimposed to variable concentrations of glutamate (in saturating glycine) that rise instantaneously and relax with a time constant of 1.2 ms. *B*, the concentration–response curve for current responses in *A* fitted to the Hill equation (Fig. 6 legend). *C*, simulated response to a train of 21 synaptic‐like glutamate stimuli that each rise to 1.1 mM and decay exponentially with a time constant of 1.2 ms. *D*, the time course for recovery from desensitization induced by a single synaptic stimulus was determined using a double pulse protocol with a variable interval. The time constant for recovery from desensitization was determined by Amplitude_2_/Amplitude_1_ = (1 − exp(−time/tau)).

Using the fitted rates, we can simulate the concentration–effect relationships for peak and steady state current response to prolonged glutamate application (Fig. 6 *C*), as well as the response to a brief synaptic‐like glutamate concentration profile. The simulated macroscopic response to 1 mM glutamate applied for 1 s had a 10–90% rise time of 5 ms. The peak and steady state concentration–effect curves predicted from these rates in response to prolonged glutamate application (in saturating glycine) had EC_50_ values of 6.5 and 2.4 μM glutamate, respectively (Fig. 6 *C*). The simulated response to brief synaptic‐like instantaneously rising and exponentially decaying glutamate concentration profiles (1.1 mM, tau 1.2 ms; Clements *et al*. 1992) had a 10–90% rise time of 4 ms (Fig. 7 *A*), compatible with NMDA receptor‐mediated synaptic whole cell currents in the mature hippocampus, cortex or cerebellar granule cells (Hestrin *et al*. 1992; Flint *et al*. 1997; Cathala *et al*. 2000; Prybylowski *et al*. 2002; Lu *et al*. 2006). The simulated synaptic response deactivated with a time course that could be best fitted by a single exponential function with a time constant of 40 ms. Varying the peak concentration of the synaptic waveform gave an EC_50_ for synaptic activation of 190 μM glutamate and a Hill slope of 1.66 (Fig. 7 *B*).

Sustained simulated application of saturating glutamate (1 mM) to excised patches for 2 s produced a macroscopic response waveform that relaxed back to a current level lower than the peak response with a variable time course. The mean response waveform from macroscopic responses (Fig. 6 *B*) to 1 mM glutamate (100 μM glycine) in nine patches was best described by a dual exponential function with a fast time constant of 119 ms (13%) and a slow time constant of 511 ms (87%). Macroscopic responses simulated using fitted rate constants from the single channel data had a similar dual exponential time course for desensitization in response to prolonged glutamate application, with a fast time constant of 151 ms (12%) and a slow time constant of 437 ms (88%). Receptor desensitization can have a significant effect on the NMDA receptor current during high frequency synaptic transmission (Tong *et al*. 1995; Popescu *et al*. 2004; Erreger *et al*. 2005). We subsequently used these fitted parameters to simulate the synaptic response to multiple synaptic‐like pulses of glutamate that instantaneously rose to 1.1 mM, and decayed to baseline exponentially with a time constant of 1.2 ms (Clements *et al*. 1992). Trains of synaptic‐like stimuli produced an incremental reduction in the synaptic current amplitude that was predicted to decrease exponentially with a time constant of tau_DESEN_ = 0.38 s to a steady state of 35% of the initial peak. This decay in synaptic peak amplitude will depend on the frequency of stimulation (Fig. 7 *C*). Evaluation of the recovery from desensitization using pairs of synaptic stimuli (Fig. 7 *D*) with a variable interval revealed a tau_RECOVERY_ of 2.1 s, compatible with the desensitization rates determined from both the single channel record and the macroscopic response time course. This desensitization and recovery time course may be neuroprotective during epileptic discharge and so is an important aspect of the receptor‐channel kinetics.

## Discussion

Current conceptual formulations of NMDA receptor function have been greatly facilitated by emerging concepts about glutamate receptor structure (Mayer, 2017). We now know that the subunits of the heterotetrameric glutamate receptors are arranged as a pair of heterodimers in a 1‐2‐1‐2 order around the ion channel for both kainate (Reiner *et al*. 2012; Paramo *et al*. 2017) and NMDA receptors (Sobolevsky *et al*. 2009; Salussolia *et al*. 2011; Riou *et al*. 2012; Karakas & Furukawa, 2014; Lee *et al*. 2014). The main result of this study is the development of a structurally constrained model of receptor activation that uniquely represents all four NMDA receptor subunits in a way consistent with the subunit composition. This model can account for single channel and macroscopic properties, and allows analysis of modification or exchange of individual subunits.

### Distinct subunit‐specific contributions to NMDA receptor activation

Each NMDA receptor subunit comprises four semi‐autonomous domains, including an amino terminal domain that is hypothesized to bind allosteric regulators, an agonist binding domain that binds glycine (GluN1) or glutamate (GluN2), a transmembrane domain that shares structural similarity to potassium channels, and an intracellular domain that provides binding sites for multiple scaffolding partners and modulatory proteins, as well as molecular tags that specify receptor trafficking (Paoletti *et al*. 2013; Wyllie *et al*. 2013; Zhou & Wollmuth, 2017; Iacobucci & Popescu, 2017). Within the context of this structural framework, explicit physical models of agonist binding, receptor desensitization, negative allosteric modulation, and concerted pore dilatation have emerged (Benveniste *et al*. 1990; Clements *et al*. 1992; Nahum‐Levy *et al*. 2001; Schorge *et al*. 2005; Talukder & Wollmuth, 2011; Hansen *et al*. 2012; Dai & Zhou, 2013; Yi *et al*. 2016; Mesbahi‐Vasey *et al*. 2017; Zhou & Wollmuth, 2017) allowing conceptualization of models of receptor function that can be constrained to be consistent with both the physical structure of the receptor and with functional data from both single channel and macroscopic current recordings.

**Figure 8:**
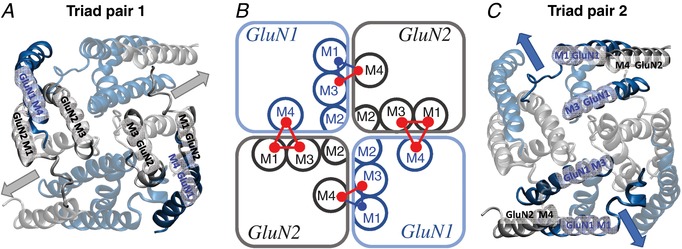
Three key motifs hypothesized to form two pairs of triads that may underlie the subunit‐dependent rates of pre‐gating conformational changes *A*, ribbon structure of the transmembrane domains and linker regions of the NMDA receptor highlighting the GluN1 subunit pre‐M4 region and GluN2 subunit pre‐M1 and M3‐SYTANLAAF and regions that we propose come together to form a pre‐gating ‘triad’ whose movement forms a rate‐limiting step in receptor activation following agonist binding and preceding pore opening. *B*, schematic diagram illustrates the four proposed NMDA receptor gating triads. Two triads are hypothesized to be dominated by the pre‐M1 and M3‐SYTANLAAF motifs of a GluN2 subunit and the GluN1 pre‐M4 (as shown in *A*). *C*, two additional triads are formed by the pre‐M1 and M3‐SYTANLAAF regions of a GluN1 subunit and the GluN2 pre‐M4. Pore dilation movements are represented by gray arrows in *A* for GluN2 subunits and blue arrows in *C* for GluN1 subunits. The pre‐M1 motif of GluN1 is hypothesized to have a reduced influence on receptor activation (and so coloured blue), given the differential effects of pre‐M1 mutations in GluN1 and GluN2 (Ogden *et al*. 2017).

For diheteromeric NMDA receptors, the alternating 1‐2‐1‐2 subunit arrangement provides a symmetry in the receptor structure (Sobolevsky *et al*. 2009; Salussolia *et al*. 2011; Riou *et al*. 2012; Karakas & Furukawa, 2014; Lee *et al*. 2014; Tajima *et al*. 2016) that we reflect in constraining the rates for two distinct conformational changes that precede rapid pore dilatation (Fig. 2). We propose that these conformational changes reflect two distinct sets of linker/TMD interactions that involve both subunits (Ogden & Traynelis, 2013; Amin *et al*. 2017; Chen *et al*. 2017; Ogden *et al*. 2017). We propose a relatively slow conformational change described by rate constants (Table 3) *k*_+s_ and *k_‐_*_s_ of 256 s^−1^ and 225 s^−1^, respectively (Figs 2 *C*, and 5 *A* and *C*) dominated by GluN2 that involves a triad comprising the GluN2 pre‐M1 helix and GluN2 SYTANLAAF region, together with GluN1 pre‐M4 linker as illustrated conceptually in Fig. 8. These three regions have conserved function and are under the strongest purifying selection in their respective proteins, as shown by the reduced variability in these regions in the healthy population (Swanger *et al*. 2016; Ogden *et al*. 2017). We assume that the rate for rearrangement of this region will be the same for both instances of it given the symmetry described above. Similarly, we propose a conformational change (rate constants *k*_+f_ and *k*_‐f_ of 4580 s^−1^ and 2500 s^−1^) that reflects rearrangement of the triad comprising the GluN1 SYTANLAAF region of M3, GluN1 pre‐M1 helix and GluN2 pre‐M4 linker (Fig. 8). We again exploit symmetry to propose that these two triads move at the same rate. Molecular dynamics simulations were made to test the hypothesis that atomic movements within the pre‐M1 helices act independently of each other. Larger domain motions involve ensembles of atomic movements and if atomic movements are not correlated this would be consistent with the assumption in the model that the pre‐gating rate constant for each subunit is independent of the state of the other subunits. A caveat to this idea is that the molecular dynamics simulations run for 1 μs while the expected lifetime of the pre‐gating states of the receptor in our model is in the order of 100 μs. Given that we cannot detect correlations with the movement of the pre‐M1 helices at short time points, it seems likely there would be less correlation at longer time periods.

We note that within this triad only GluN1 SYTANLAAF and GluN2 pre‐M4 are under purifying selection, with apparently less functional contribution of GluN1 pre‐M1 (Ogden *et al*. 2017), which may amplify the role of the GluN2 pre‐M4 (Amin *et al*. 2017). The implementation of this symmetry for diheteromeric receptors will allow a logical progression to analysis of triheteromeric receptor function where the GluN2 symmetry of subunit structure is lost (Lu *et al*. 2017) and therefore any functional model for triheteromeric receptors must be able to allow the rates for the gating arrangements dominated by individual subunits to differ.

In Fig. 6 we show that using rates derived from single channel fitting, the macroscopic properties of diheteromeric GluN1/GluN2A receptors can be accurately reproduced by mechanisms incorporating transitions representing subunit‐dependent clamshell closure around the agonist, unique subunit‐dependent conformational changes that we hypothesize reflect movement of interacting linker regions that precede gating (e.g. Tajima *et al*. 2016), and a dimer‐dependent desensitization reflecting a conformational change at the interface between the GluN1‐GluN2 agonist binding domains (Furukawa *et al*. 2005; Alsaloum *et al*. 2016). These mechanisms are also consistent with data describing unique roles of the linker regions in gating (Kazi *et al*. 2013, 2014; Ogden & Traynelis, 2013; Ogden *et al*. 2017; Chen *et al*. 2017; Zhou & Wollmuth, 2017). Our macroscopic current recordings (Fig. 6) show that GluN2A receptors respond to rapid application of glutamate with a current that rises to a peak open probability of about 0.5 with a 10–90% rise time that is less than 10 ms. Following rapid removal of glutamate, the current response deactivates with a time constant of about 40 ms (Wyllie *et al*. 1998; Vicini *et al*. 1998; Erreger & Traynelis, 2005; Zhang *et al*. 2008). During prolonged agonist application (Fig. 6) in dialysed outside‐out patches GluN2A receptors desensitize to a steady state level that is approximately 20% of the peak current, which we assume involves the agonist binding domain dimer interface, given the strong precedent established in AMPA receptors (Sun *et al*. 2002).

### Interactions between subunits

A key feature of the twofold symmetry in the extracellular domain of the NMDA receptors is the arrangement of a pair of glycine‐binding GluN1 subunits and two glutamate‐binding GluN2 subunits (GluN2A–D). The GluN2 subunit is well known to influence receptor properties, including the time course of NMDA receptor‐mediated synaptic transmission, agonist potency, Mg^2+^ sensitivity, Ca^2+^ permeability, single channel conductance and open probability (Erreger *et al*. 2004; Cull‐Candy & Leszkiewicz, 2004; Yuan *et al*. 2009; Traynelis *et al*. 2010; Paoletti *et al*. 2013; Wyllie *et al*. 2013; Glasgow *et al*. 2015; Iacobucci & Popescu, 2017). This variation in receptor properties is exploited at different developmental stages and in different brain regions to endow synapses with specific properties. The rapidly deactivating GluN2A subunit shortens the window for integration of synaptic activity and shifts synaptic signalling properties towards more rapid and high fidelity signalling (Flint *et al*. 1997; Lu *et al*. 2001; Carmignoto & Vicini, 1992; Hestrin, 1992; Markram *et al*. 2012; Wyllie *et al*. 2013). The model we present here provides a mechanistic basis (represented by *k*_+s_ and *k*_‐s_ rate constants) to understand the differences in properties observed between diheteromeric receptors containing different GluN2 subunits (Erreger *et al*. 2005, 2007). However, it remains unclear whether this form of model will reproduce intriguing interactions between GluN2 subunits, such as the GluN2A domination of the response time course of triheteromeric GluN1/GluN2A/GluN2B NMDARs or variations in glycine affinity (Hansen *et al*. 2014; Sun *et al*. 2017).

### Influence of subunit‐dependent pre‐gating transition rates on agonist affinity

It is well established that there is a negative cooperativity between glutamate and glycine binding (Mayer *et al*. 1989; Vycklicky *et al*. 1990; Benveniste & Mayer, 1991; Nahum‐Levy *et al*. 2001), and there is strong evidence for allosteric interactions between the binding of N‐terminal domain ligands and agonist affinity (Zheng *et al*. 2001; Erreger & Traynelis, 2008). Therefore, it is quite likely that interactions at the extracellular subunit interfaces transmit allosteric effects between subunits. The current form of model includes dimer‐dependent desensitization and could also represent agonist binding domain–dimer interactions, but not allosteric amino terminal domain–agonist binding domain interactions. We explored the influence of receptor pre‐gating (*k*_s_ and *k*_f_ rate constants) on glutamate and glycine macroscopic affinity by simulations using the set of rate constants we derived from fitting single‐channel and macroscopic currents. In the absence of glycine the channel cannot open, and the receptor mechanism can only visit a small subset of the states illustrated in Fig. 6 *A*: those states where the GluN1 subunits have not undergone pre‐gating. The calculated macroscopic glutamate binding affinity for the receptor will therefore depend only on the values of the *k*_+A_, *k*_‐A_ and *k*_+s_ and *k*_‐s_ rate constants. The macroscopic EC_50_ for glutamate binding under these conditions is 1.8 μM. In the presence of saturating glycine where the mechanism can visit all states shown in Fig. 6 *A*, the glutamate steady state EC_50_ is 3.0 μM, showing that at least a portion of the glutamate–glycine allosteric interactions could reflect availability of pre‐gating states following binding of both glutamate and glycine.

It remains unknown whether a GluN2 pre‐gating step might take place only after the partner GluN1 has bound glycine, for example. Our current model (Fig. 6) has subunit gating steps that are independent, but our working hypothesis that gating triads (Fig. 8) are the structural correlate of these pre‐gating steps suggests elements of GluN1 and GluN2 influence each pre‐gating step, even if a single subunit may dominate the rate. Excised outside‐out patches from HEK cells expressing GluN1/GluN2A that are bathed in maximal glutamate (100 μM) and activated by rapidly applying glutamate and glycine (1 mM) produce current responses that activate at the same speed (10–90% rise time 9.3 ± 0.5 ms, *n* = 3, data not shown) as patches activated by glutamate described above (Table 2). This result argues against the idea that the slower GluN2‐dependent pre‐gating step can occur without glycine binding, because if they could, one would predict a faster rise time of the receptor response dominated by *k*_+f_ . Thus, additional experimental work remains in order to understand the exact role of glycine binding in NMDA receptor activation.

### Receptor desensitization

Desensitization describes the process whereby a response is decreased in the continued presence of a stimulus. Sun *et al*. (2002) proposed that a physical rearrangement of the dimer interface that exists between agonist binding domains represents a model for desensitization for AMPA receptors (Meyerson *et al*. 2014), and there is evidence to suggest that NMDA receptors share mechanistic features of desensitization with AMPA receptors (Furukawa *et al*. 2005; Alsaloum *et al*. 2016) in addition to kinetic influences from both N‐terminal and C‐terminal (Iacobucci & Popescu, 2017) domains. Although we still do not know the physical basis for desensitization in NMDA receptors, the long‐lived closed periods that are very evident in single channel recordings (Fig. 1 *A*) are considered to represent ‘desensitized’ receptor states. There are several structural precedents that could explain the existence of these long‐lived closed states, and thus we retain the nomenclature of desensitized states. Following the precedent established in the AMPA receptor literature (Sun *et al*. 2002; Meyerson *et al*. 2014), we have explicitly modelled transitions to these desensitized states as closed states that occur when all four subunits have bound agonist and both GluN1 and GluN2 subunits within at least one heterodimer are activated, as opposed to when only four subunits in the receptor have completed pre‐gating. When the equivalent model to that in Fig. 5 *C* (but with entry to a single desensitized state only possible after all four subunits have competed pre‐gating) is fitted to our data set, the log‐likelihood for this model is much less favourable (likelihood ratio test *P* < 0.001, d.f. = 16). Thus, inclusion of explicit desensitization steps allows the macroscopic model to reproduce properties of agonist‐dependent desensitization (Figs 6 and 7) and correctly account for the macroscopic agonist affinity (Fig. 6). We recognize that the data we use to evaluate desensitization are from dialysed patches, which show greater desensitization (Sather *et al*. 1990) even though deactivation is largely unchanged by patch excision (Lester *et al*. 1990; Lester & Jahr, 1992).

### Differential subunit contributions to receptor activation

While we have assumed non‐cooperativity between subunits, there is ample evidence to show that each GluN2 subunit within the triheteromeric GluN1/GluN2A/GluN2B receptor contributes to the functional properties of these receptors. Specifically, triheteromeric receptors display unique macroscopic properties that are different from those of either diheteromeric receptor (Hansen *et al*. 2014; Stroebel *et al*. 2014; Sun *et al*. 2017). Additionally, similar observations of unique properties for receptors with non‐equivalent GluN2 subunits have been made for diheteromeric receptors with one subunit containing a point mutation (e.g. Yuan *et al*. 2014; Li *et al*. 2016; Swanger *et al*. 2016; Ogden *et al*. 2017). A particularly striking example involves *de novo* mutations at a proline residue within the pre‐M1 helix of the agonist binding domain‐TMD linker in GluN1, GluN2A and GluN2B (Ogden *et al*. 2017). For GluN2, but not GluN1, mutation of this proline to an arginine produces an incremental slowing of the deactivation rate, dependent on the number of mutant subunits in the tetrameric complex. In addition, the receptor macroscopic current rise time and single channel conductances change only when two copies of the mutant GluN2 subunit are present. With two mutant copies, the response to a brief pulse of glutamate continues to rise slowly, long after the glutamate has been removed, which indicates that these effects involve changes to the pre‐gating steps rather than agonist binding (Ogden *et al*. 2017). Based on these two independent but related concepts, we characterized triheteromeric GluN1/GluN2A/GluN2B receptors (Fig. 3) containing the GluN2A‐P552R mutation or both GluN2A‐P552R and GluN2B‐P553R mutations. Our results showed that the GluN2A‐P552R/GluN2B receptor responses show a deactivation time course that is slowed by approximately 7‐fold, but retained the wild type‐like rise time. This result is consistent with the effects of the P552R mutation on diheteromeric receptors, and supports our hypothesis (as represented in the mechanisms shown in Figs 5 and 6) that channel opening can occur when only one of the two GluN2 subunits have completed pre‐gating.

### Relation to other mechanisms describing pre‐gating transitions in channel activation

Finally, this model captures many shared features of other models that can successfully reproduce the kinetics of ligand‐gated ion channel activation. For example, a linear reaction scheme with three adjacent closed states and two adjacent open states (C‐C‐C‐O‐O model) is able to reproduce a surprisingly wide range of diheteromeric GluN1/GluN2A channel properties when data within clusters (disregarding agonist binding and desensitized states) are analysed (Auerbach & Zhou, 2005; Erreger *et al*. 2005; Iacobucci & Popescu, 2017), and elements of this model could be captured in a portion of our grid model along the vector representing pre‐gating conformational changes. Addition of agonist binding and desensitized states allows a linear model to reproduce the time course of both synaptic and macroscopic currents (Lester & Jahr, 1992; Iacobucci & Popescu, 2017) without invoking subunit‐dependent properties. Likewise, the concept of subunit pre‐gating states has been applied successfully to tetrameric models of the cyclic nucleotide gated (CNG) channels (Ruiz & Karpan, 1999) and the BK Ca^2+^ activated potassium channels (reviewed in Magleby, 2003). As with our NMDA receptor model, the BK and CNG channel models also included the possibility of channel opening when three of the four subunits have undergone pre‐gating. In contrast, a pre‐gating model that successfully describes the pentameric glycine receptors (Burzomato *et al*. 2004) has three pre‐gating steps, so the model does not directly relate gating to conformational changes in individual receptor subunits. Likewise, NMDA models (e.g. C‐C‐C‐O‐O) that include pre‐gating steps in a linear sequence are not subunit related (e.g. Fig. 4 of Iacobucci & Popescu, 2017). Models describing NMDA receptor activation that include gating cycles with fast and slow steps and agonist binding (Banke *et al*. 2003; Erreger *et al*. 2005; Schorge *et al*. 2005; Wyllie *et al*. 2006) are also captured in the subunit specific models investigated here. Thus, we view our approach as entirely compatible with previously described mechanisms that gave high‐precision matches to single channel data sets, while in addition, here we incorporate key subunit‐dependent features of current structurally derived models of NMDA receptors.

## Appendix

**Figure A1:**
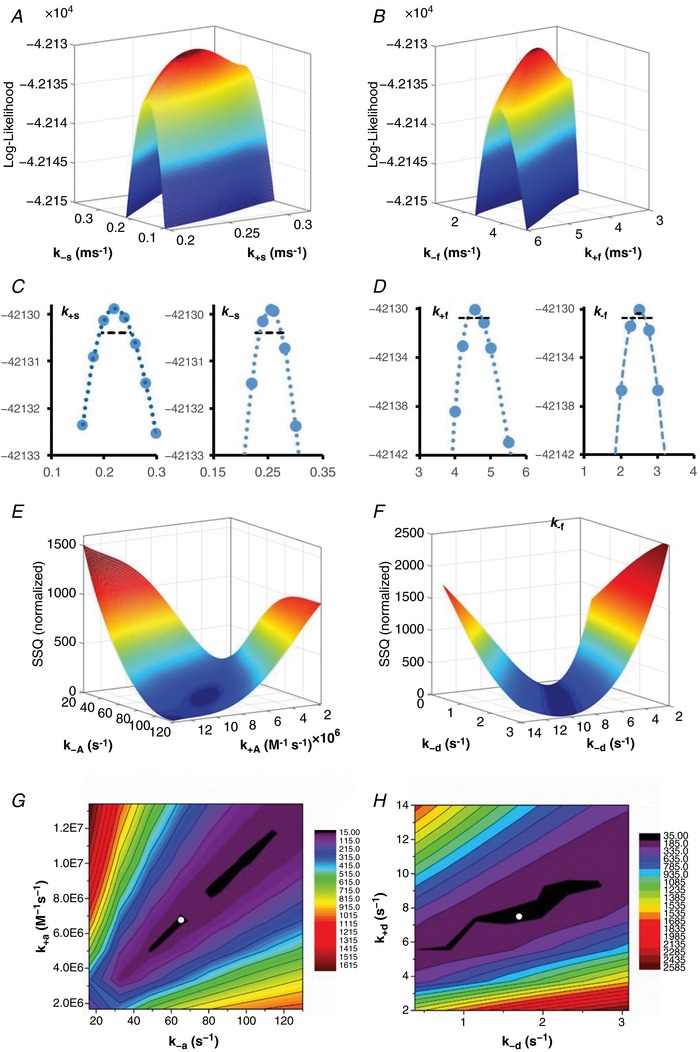
Sensitivity analysis of model rate constants *A* and *B* demonstrate how the likelihood surface around the maximum depends on the value of the rate constants *k_+_*_s_ and *k_‐_*_s_ (*A*) and *k_+_*_f_ and *k_‐_*_f_ (*B*). *C* and *D* illustrate estimation of the 0.5 unit log‐likelihood intervals for *k_‐_*_s_, *k*_+s_, *k_‐_*_f_ and *k*_+f_, respectively. For each panel, filled symbols show the best‐fit log‐likelihood found when the data set are fitted with the parameter fixed at the values shown on the *x*‐axis. Dashed lines show the shape of a cubic polynomial used to interpolate the value of the parameter corresponding to a log‐likelihood of 0.5 units less than the maximum. The peak of each curve corresponds to the best‐fit value of the rate constant (*k_‐_*_s_ = 220 s^−1^, *k*_+s_ = 254 s^−1^, *k*_‐f_ = 4550 s^−1^, *k*_+f_ = 2490 s^−1^) and the values of the rate constant giving a log‐likelihood value of 0.5 less than the maximum were then found numerically and are indicated by horizontal dashed lines. These were; *k*_‐s_ = 187 s^−1^ and 258 s^−1^; *k*_+s_ = 227 s^−1^ and 282 s^−1^; *k*_‐f_ = 4379 s^−1^ and 4709 s^−1^; *k*_+f_ = 2403 s^−1^ and 2561 s^−1^. *E* and *F* illustrate how the sum of squares (SSQ) obtained from fitting macroscopic current data depends on the values of the glutamate association (*k*_+A_) and dissociation (*k*_‐A_) rates, and the rates for the onset (*k*_+d_) and recovery (*k*_‐d_) from desensitization. *G* and *H* show the surface contour for the SSQ landscape with the minima indicated by a white dot.

A sensitivity analysis was performed for the model rate constants estimated by fitting single channel and macroscopic data. In the case of single channel data, this allows an investigation of the sensitivity of the shape of the likelihood surface near the maximum to small changes in a single parameter. For rate constants estimated by least squares fitting of macroscopic currents, the sensitivity of the sum of squares can be determined for small changes in parameter values around the best‐fit value for the glutamate binding (*k*_+A_) and unbinding (*k*_‐A_) rates, and rates for desensitization (*k*_+d_, *k*_−d_). The results are illustrated for the *k*_s_ and *k*_f_ rate constants in Fig. A1 *A*–*D* and for *k*_+d_, *k*_−d_, *k*_+A_ and *k*_−A_ in Fig. A1 *E–H*.

The values of *k*_+s_ and *k_‐_*_s_ (Fig. A1 *A*) and *k*_+f_ and *k_‐_*_f_ (Fig. A1 *B*) were modestly correlated with the likelihood approaching a single peak at the best‐fit values of the parameters while for *k*_+A_, *k*_−A_ and for *k*_+d_, *k*_−d_ the best‐fit values are at the minimum of a valley in the sum of squares surface. Figure A1 *C* and *D* illustrates the estimation of likelihood intervals for *k*_+s_ and *k*_−s_ (Fig. A1 *C*) and *k*_+f_ and *k*_−f_ (Fig. A1 *D*). Likelihood intervals were estimated by fixing the value of the test rate constant at the value shown on the *x*‐axis and maximizing the value of the log‐likelihood with respect to the other rate constants in the mechanism (Colquhoun & Sigworth, 1995). These data demonstrate that the estimated rate constants were well determined by the fitting process *k*_+s_ = 256 s^−1^ (0.5 unit likelihood interval range 227–282 s^−1^) and *k*_−s_ = 225 s^−1^ (187–258 s^−1^) and *k*_+f_ = 2500 s^−1^ (2403–2561 s^−1^) and *k*_−f_ = 4550 s^−1^ (4379–4709 s^−1^).

The least squares fitting of the model to the macroscopic waveforms in Fig. 6 yielded a glutamate association rate constant *k*_+A_ of 6.7 × 10^6^ M^−1^ s^−1^ and a dissociation rate constant *k*_−A_ of 65 s^−1^, which yielded a sum of squares difference of 5.51; this value is unitless because it was calculated from the waveforms normalized to the maximal peak current. A sensitivity analysis was conducted over a range of rate constants, with *k*_+A_ values ranging from 1.68 × 10^6^ to 1.34 × 10^7^ M^−1^ s^−1^ and *k*_−A_ values ranging from 16.25 to 130 s^−1^. The sum of squared differences (SSQ) was calculated from the simulated waveform and recorded mean waveform for each possible combination of rates for both waveforms shown in Fig. 6 (Fig. A1 *A*). The best fit of the model in Fig. 6 to the macroscopic waveforms yielded onset of and recovery from desensitization rates *k*_−d_ of 1.54 s^−1^ and *k*_+d_ of 7.41 s^−1^, respectively. Rates of onset of and recovery from desensitization were also co‐varied and the sum of squares difference determined for all combinations. *k*_−d_ was varied between 0.385 and 3.08 s ^−1^ and *k*_+d_ was varied between 1.85 and 14.8 s^−1^. Figure A1 *E* and *F* plots the sum of squares for each co‐varied pair of rates for association and dissociation rate constants (Fig. A1 *E*), with desensitization rates held constant to those determined from the best fit. Similarly, the onset and recovery of desensitization were co‐varied, with association and dissociation held constant to those determined from the best fit (Fig. A1 *F*). The sensitivity analysis confirms the presence of a single minimum in the least squares surface generated when fitting the model in Fig. 6. However, Fig. A1 *G* and *H* reveals that the relation between the SSQ and parameter values has a shallow basin with minimum value (shown as a white circle) indicating the fit will give similar values for the glutamate equilibrium dissociation constant (*k*_−A_/*k*_+A_) along the floor of the basin.

### Comparison of fitted models with different paths to the open state

**Figure A2:**
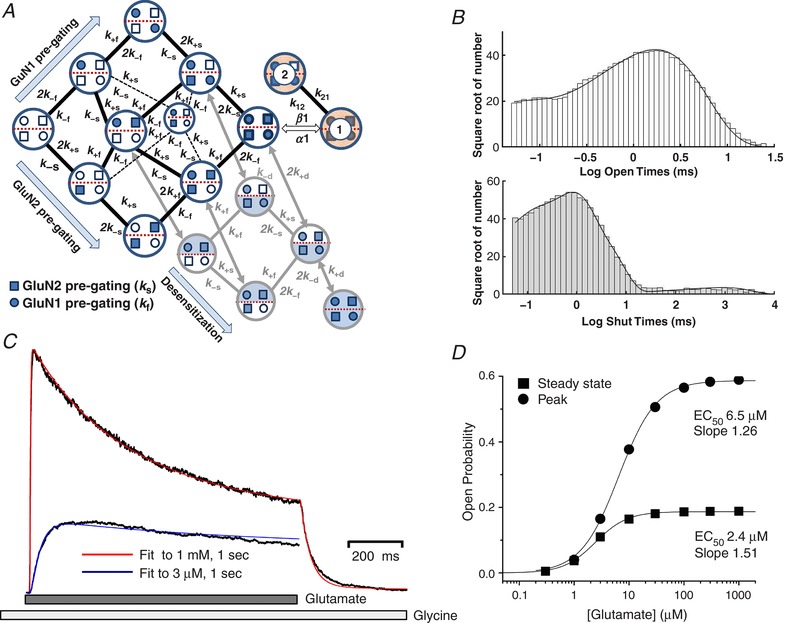
Evaluation of a model that can only reach the open state when all four pre‐gating steps have occurred *A*, model that is identical to that in Fig. 5, except that channel opening can only occur from a state with all four activated pre‐gating steps. For clarity, not all rate constants are shown for desensitizing steps. *B*, super‐position of predicted open and closed duration histograms from a maximum likelihood fit of the model in *A* to the idealized data. *C*, macroscopic curves were the same as in Fig. 6, except that they were fitted by the model in *A* with glutamate binding sites added as described for Fig. 6. *D*, predicted peak (filled circles) and steady state (filled squares) *P*_open_ curves.

We compared the model determined in Figs 5 and 6 with a virtually identical model in which opening can only occur when all four (not 3) pre‐gating transitions have occurred (Fig. A2). While this model can produce a good fit to the single channel data, the log‐likelihood for this model was significantly less favourable, and the pre‐gating rate constants were slower (Table A1). These data suggest that our representation of activation when either three or four pre‐gating steps have occurred is more compatible with the data. Fitting this model to the same macroscopic current response waveforms (Fig. A2) produced an accurate fit, but with different glutamate association and dissociation rates. Using the fitted rates, we can simulate the concentration–effect relationships for peak and steady state current response to prolonged glutamate application, as well as the response to the brief synaptic glutamate concentration profile. The peak and steady state concentration–effect curves predicted from these rates in response to prolonged glutamate application (in saturating glycine) had EC_50_ values of 6.5 (peak) and 2.5 μM glutamate (steady state). Sustained simulated application of saturating glutamate (1 mM) to excised patches for 3 s produced a rapidly activating (10–90% rise time 5 ms) macroscopic response waveform that relaxed back to a current level lower than the peak response with a time course that can be described by a dual exponential with a fast time constant of 146 ms (10%) and a slow time constant of 423 ms (90%). The simulated response to a synaptic‐like instantaneously rising exponentially decaying (1.1 mM, tau 1.2 ms; Clements *et al*. 1992) glutamate concentration profile had a 10–90% rise time of 4 ms and deactivated with a time course that was best fitted by a single exponential function with time constant of 37 ms, compatible with the model in Fig. 6. Varying the peak synaptic glutamate concentration gave an EC_50_ value for synaptic activation of 201 μM glutamate and a Hill slope of 1.32. While the parameters determined from a model where opening of the channel only occurs following all four pre‐gating steps can reproduce some features of NMDA receptor macroscopic activation, we favour the model with two distinct paths to the open state (Figs 5 and 6) because it logically accounts for the rise time dependence on number of mutant subunits (Fig. 3), allows for correlations in open times, produces a better fit to the single channel data, and yields a glutamate EC_50_ that matches experimental values.

**Table A1:** Comparison of rate constants from models with different paths to the open state

	Single channel fit	Macroscopic fit	Single channel fit	Macroscopic fit
	model (Figs 5 and 6)	model (Figs 5 and 6)	model (Fig. A2)	model (Fig. A2)
*k*_12_	1390	*1390*	15000	*15000*
*k*_21_	15200	*15200*	4110	*4110*
α_1_	344	*344*	3700	*3700*
β_1_	4130	*4130*	4010	*4010*
α_2_	5670	*5670*	—	—
β_2_	3550	*3550*	—	—
*k*_+f_	4580	*4580*	4880	*4880*
*k*_−f_	2500	*2500*	2730	*2730*
*k*_+s_	256	*256*	572	*572*
*k*_−s_	225	*225*	87.3	*87.3*
*k*_+d_	5.28	**7.41**	3.32	**4.63**
*k*_−d_	1.78	**1.54**	1.81	**1.60**
*k*_+A_	—	**6.7 × 10^6^**	—	**1.3 × 10^7^**
*k*_−A_	—	**65**	—	**483**
LL	−42130	—	−42144	—

All rate constants in s^−1^ except *k*_+A_, which is in M^−1^ s^−1^. Rates were estimated from composite fit of all patch data given to 3 significant figures. The total number of open–closed transitions was 108,467; LL is the log likelihood. Rates shown in italics were fixed during macroscopic fitting so the number of free parameters for macroscopic fits was 4 (shown in bold), while in the single channel fits there were 11 (*k*_12_ is determined by microscopic reversibility) free parameters for the model used in Figs 5 and 6, and A2 for the model in Fig. A2.

## Additional information

### Competing interests

S.F.T. is a co‐founder of NeurOp Inc., a PI on a research grant from Janssen to Emory University, and a member of the Scientific Advisory Board for Sage Therapeutics.

### Author contributions

K.K.O., M.J.M. and K.M.V. conducted experiments at Emory University. K.K.O. implemented the missed event fitting in MATLAB. S.A.K., P.B. and C.B. performed molecular dynamics simulations and analyses. D.C.L. contributed to interpretation of molecular dynamics data. K.K.O., M.J.M., S.F.T., S.A.K. and A.J.G. performed data analysis. A.J.G. and S.F.T. performed simulations. All authors contributed to writing the manuscript. All authors have approved the final version of the manuscript and agree to be accountable for all aspects of the work. All persons designated as authors qualify for authorship, and all those who qualify for authorship are listed.

### Funding

This work was supported by the National Institute of Neurological Disorders and Stroke (NINDS, NIH) (NS036654, NS065371; S.F.T.), the Wellcome Trust (A.J.G.) and the BBSRC (A.J.G.).
